# Structure and dynamics of the *Arabidopsis O*-fucosyltransferase SPINDLY

**DOI:** 10.1038/s41467-023-37279-1

**Published:** 2023-03-20

**Authors:** Shivesh Kumar, Yan Wang, Ye Zhou, Lucas Dillard, Fay-Wei Li, Carly A. Sciandra, Ning Sui, Rodolfo Zentella, Emily Zahn, Jeffrey Shabanowitz, Donald F. Hunt, Mario J. Borgnia, Alberto Bartesaghi, Tai-ping Sun, Pei Zhou

**Affiliations:** 1 Department of Biochemistry, Duke University School of Medicine, Durham, NC 27710 USA; 2 Department of Biology, Duke University, Durham, NC 27708 USA; 3 Department of Computer Science, Duke University, Durham, NC 27705 USA; 4 Genome Integrity and Structural Biology Laboratory, National Institute of Environmental Health Sciences, Research Triangle Park, NC 27709 USA; 5 Plant Biology Section, Cornell University, Ithaca, NY 14853 USA; 6 Boyce Thompson Institute, Ithaca, NY 14853 USA; 7 Department of Chemistry, University of Virginia, Charlottesville, VA 22904 USA; 8 Department of Pathology, University of Virginia, Charlottesville, VA 22903 USA; 9 Department of Electrical and Computer Engineering, Duke University, Durham, NC 27708 USA

**Keywords:** Cryoelectron microscopy, Plant sciences

## Abstract

SPINDLY (SPY) in *Arabidopsis thaliana* is a novel nucleocytoplasmic protein *O*-fucosyltransferase (POFUT), which regulates diverse developmental processes. Sequence analysis indicates that SPY is distinct from ER-localized POFUTs and contains N-terminal tetratricopeptide repeats (TPRs) and a C-terminal catalytic domain resembling the *O*-linked-*N*-acetylglucosamine (GlcNAc) transferases (OGTs). However, the structural feature that determines the distinct enzymatic selectivity of SPY remains unknown. Here we report the cryo-electron microscopy (cryo-EM) structure of SPY and its complex with GDP-fucose, revealing distinct active-site features enabling GDP-fucose instead of UDP-GlcNAc binding. SPY forms an antiparallel dimer instead of the X-shaped dimer in human OGT, and its catalytic domain interconverts among multiple conformations. Analysis of mass spectrometry, co-IP, fucosylation activity, and cryo-EM data further demonstrates that the N-terminal disordered peptide in SPY contains *trans* auto-fucosylation sites and inhibits the POFUT activity, whereas TPRs 1–5 dynamically regulate SPY activity by interfering with protein substrate binding.

## Abstract (web summary)

Kumar and colleagues report cryo-EM structures of *Arabidopsis* *O*-fucosyltransferase SPINDLY in its apo and GDP-fucose bound states, revealing distinct active-site features enabling GDP-fucose recognition and surprisingly dynamic conformations that regulate its enzymatic activity.

## Introduction

Until recently, protein *O*-fucosylation of serine or threonine residues, carried out by the ER-localized protein *O*-fucosyltransferases (POFUTs), has only been found in secreted or cell surface proteins^1,2^. The discovery of the novel nucleocytoplasmic POFUT, SPINDLY (SPY) in *Arabidopsis thaliana* (*At*) that modifies the nuclear transcription regulators DELLAs^3^ together with the identification of a variety of intracellular substrates of SPY^4–6^ and of the SPY ortholog in the parasitic protist *Toxoplasma gondii*^7,8^ have dramatically expanded the functional realm and biological implication of protein *O*-fucosylation.

SPY was initially discovered through recessive mutations that conferred resistance to the gibberellin (GA) biosynthesis inhibitor paclobutrazol^9^. Further studies showed that SPY regulates plant development during both vegetative and reproductive stages by modulating multiple hormone signaling activities, light response, and circadian signaling^10–12^. Molecular cloning and sequence analysis of SPY^13^ show that it encodes 11 tetratricopeptide repeats (TPRs) and a C-terminal catalytic domain resembling OGTs of the Glycosyltransferase Family 41 (GT41)^14,15^, leading to the initial designation of SPY as an OGT^16^, though the predicted enzymatic activity was never detected in vitro. Recently, through detailed mass spectrometry analysis, in vitro enzymatic assays, and genetic analysis, we have discovered SPY as a novel POFUT^3^, whereas its paralog SECRET AGENT (SEC) is an OGT^17^. SPY is highly selective for transferring the *O*-fucose monosaccharide from GDP-fucose to the Ser and Thr residues of a variety of signaling proteins, including the nuclear transcription regulators DELLAs^3^, the circadian clock component PSEUDO-RESPONSE REGULATOR 5 (PRR5)^4^, the RNA splicing factor ACINUS^6^, and the chloroplast-localized co-chaperonin CPN20^5^. The TPR domain of SPY interacts with protein substrates (e.g., PRR5), and an in-frame deletion within TPRs 9–10 in the spy*-8* mutant^18^ abolishes this interaction^4^, suggesting that TPRs’ function is to recruit protein substrates.

Despite its POFUT activity, sequence analysis of SPY shows it is distinct from ER-localized POFUTs of GT65 and GT68 families^3,19^. Additionally, the cytoplasmic and nuclear distribution of SPY resembles OGTs^20^. Here we report the cryo-EM structures of SPY and its complex with GDP-fucose, unveiling the molecular basis of its substrate specificity. SPY forms an antiparallel dimer, which is distinct from the X-shaped dimer of TPR-containing OGTs^21^. We show that SPY dynamically cycles through multiple conformations (inward, middle, and outward), with the catalytic domain moving and rotating along the TPRs. In particular, the catalytic domain in the “middle” state is held together by the N-terminal TPRs, whereas the inward and outward states are observed when the EM densities of the N-terminal TPRs are not observed. Unexpectedly, we found that the disordered peptide N-terminal to TPRs is *trans* auto-fucosylated and inhibits SPY activity, whereas deletion of TPRs 1–5 dramatically enhances its binding affinity toward the RGA substrate, implicating a regulatory role of the N-terminal peptide and TPRs 1–5.

## Results

### Cryo-EM structure of full-length SPY

**Fig. 1:**
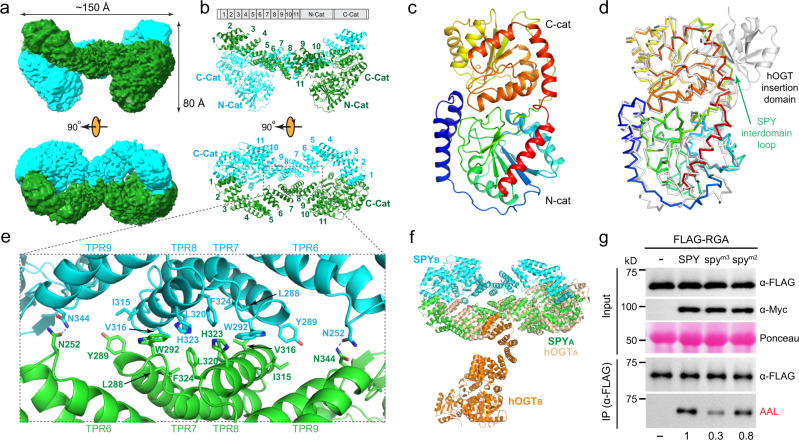
*Arabidopsis* SPY forms an antiparallel dimer. **a** Cryo-EM density map of SPY from the side and top views, with individual monomers colored in green and cyan. **b** Ribbon diagram of the SPY dimer, revealing all 11 predicted TPRs, the catalytic domain, and an overall dimer arranged in the shape of figure “8”. The individual TPRs and the N-terminal half (N-Cat) and C-terminal half (C-Cat) of the catalytic domain are labeled. **c** Catalytic domain of SPY is shown in the cartoon model and colored in rainbow with the N-terminus in blue and C-terminus in red. **d** Overlay of the SPY catalytic domain (rainbow) with the hOGT catalytic domain (gray; PDB 3PE3). The SPY interdomain loop connecting the N-Cat and C-Cat and the hOGT insertion domain are labeled. **e** The dimeric interface of SPY formed by antiparallel packing of TPRs 6–9. Interfacial residues are shown in the stick model and are labeled. **f** SPY and hOGT have dissimilar dimeric interfaces. Subunits of SPY and hOGT (PDB 7NTF^21^) are labeled. **g** Mutations in SPY disrupting its dimerization caused reduced POFUT activities in planta. FLAG-RGA proteins were transiently expressed alone (−) or co-expressed with Myc-SPY or spy mutant proteins (m2: L320A and F324A, m3: W292A, L320A, F324A) in *Nicotiana benthamiana*. Top panel (Input): Immunoblots containing total protein extracts were probed with either α-FLAG or α-Myc. The image of the Ponceau-stained gel blot shows similar loading. Bottom panel (α-FLAG IP’ed samples): Immunoblots containing affinity-purified FLAG-RGA proteins were probed with either α-FLAG to show even loading of FLAG-RGA proteins or AAL-biotin followed by HRP-streptavidin to detect *O*-fucosylated FLAG-RGA. The activity of WT SPY was set as 1. –, not detectable. Representative images of three biological repeats are shown. Source data are provided as a Source Data file.

Recombinant full-length SPY (101.4 kDa) was expressed and purified from insect cells. Multiple conformations of the SPY dimer could be resolved using cryo-EM, with overall resolutions varying from 3.6 to 3.9 Å (Supplementary Fig. 1a, b and Supplementary Table 1). In the symmetric dimeric state containing the most complete structural features, the entire predicted 11 TPRs and the catalytic domain are visible in the EM density (Fig. 1a, b). Viewing from the side, each monomer resembles a swimming tadpole, with the N-terminal TPRs forming the curly tail and the C-terminal catalytic domain forming the head. Viewing from the top, two tadpoles come together in a head-to-tail fashion to form an overall shape of figure “8”, with each tadpole occupying an evenly divided figure “8” along the long axis (Fig. 1a, b).

The catalytic domain of SPY adopts the classical glycosyltransferase-B (GT-B) fold^22,23^ (Fig. 1c). The N-terminal half of the catalytic domain (N-Cat) has a central, seven stranded β-sheet surrounded by six helices, whereas the C-terminal half of the catalytic domain (C-Cat) contains a well-conserved β/α/β Rossmann-fold^24^. The catalytic domain of SPY (R431-K846) overlays very well with the catalytic domain of human *O*-GlcNAc transferase (hOGT, H496-K1028; PDB 3PE3^25^) of the same GT41 family (http://www.cazy.org/GT41.html), with an overall backbone RMSD of 1.3 Å (Fig. 1d). However, the N-Cat and C-Cat of SPY are connected by an extended loop, whereas this interdomain loop is replaced in hOGT with an insertion domain consisting of seven strands and four helices (Fig. 1d).

Located N-terminal to the catalytic domain are 11 TPRs that form an extended, right-handed supercoil (Fig. 1b). The TPR is a well-characterized structural motif consisting of ~34 residues forming two antiparallel helices in each repeat unit^26^. TPRs 6, 7, 8, and 9 from one subunit interact with TPRs 9, 8, 7, and 6 from the other subunit to form an extensive interface of the SPY dimer (Fig. 1e). At the center of the dimeric interface lie a set of hydrophobic and aromatic residues, including L288, Y289, W292, L320, H323, and F324 from TPR7 and TPR8. These interactions are buttressed by peripheral polar interactions between N252 of TPR6 and N344 of TPR9. Most unexpectedly, the N-terminal TPRs from one subunit curl around and touch down on the surface of the C-terminal catalytic domain from the opposite subunit through TPR1 (Fig. 1a, b). Despite the limited local resolution of TPR1, it is evident that such an interaction minimally involves Y49 and R56 of the first helix of TPRs and surface exposed hydrophilic (R685, D717) and hydrophobic residues (F654) of the C-Cat (Supplementary Fig. 2).

The feature of TPRs touching down on the catalytic domain is unique in the SPY family of POFUTs, as no other POFUTs possess TPRs^1,2^. In contrast, the TPRs in mammalian OGTs, such as the human OGT, form an X-shaped dimer, generating a large gap between the N-terminal TPRs and the C-terminal catalytic domain either within the subunit or across the subunits^21^ (Fig. 1f). Human OGT and SPY also do not use the same TPRs for the dimer formation such that when dimeric human OGT and SPY are superimposed on their first subunits, their second subunits are located on two opposite sides of the first subunits (Fig. 1f).

Intriguingly, many of the recessive mutations at the *SPINDLY* (SPY) locus that partially rescued the dwarf phenotype in the GA-deficient mutant *ga1* are clustered in TPRs 7, 9, and 10^18^. spy*-1*, spy*-2*, and spy*-8* result in a deletion of M354-Q376 in TPR9–10; spy*-7* causes I390F and deletion of L391-A392 in TPR10; and spy*-6*, spy*-9*, spy*-10*, and spy*-11* have G268 in the TPR7 mutated to either glutamate or arginine. As these mutations likely disrupt the folding of one or more TPRs, these genetic studies support the notion that the TPRs play an important role in regulating the SPY function.

We investigated whether the dimeric architecture contributes to the SPY function in planta by generating alanine substitutions of TPR residues, W292, L320, and F324, at the dimer interface. We found that both the W292A/L320A/F324A *spy*^*m3*^ triple mutation and the L320A/F324A *spy*^*m2*^ double mutation shifted the mutant protein elution volume to 12.7 mL from the elution volume of 11.9 mL of WT SPY on the Superdex 200 increase 10/300 GL column, suggesting that alanine mutations of these structurally observed interfacial residues compromised the formation of the SPY dimer (Supplementary Fig. 3). We then compared the catalytic activities of WT and mutant spy proteins in planta using the transient co*-*expression assays in *N. benthamiana* by agroinfiltration. FLAG-tagged RGA, an *Arabidopsis* DELLA protein, was used as the protein substrate in these enzyme assays. The relative levels of *O-*fucosylation in the FLAG-RGA protein in different samples were examined by affinity purification of FLAG-RGA proteins and protein blot analysis using a terminal fucose-specific lectin (*Aleuria aurantia* lectin, AAL)^8^. Even though the WT SPY and the spy^m2^ and spy^m3^ proteins were expressed at similar levels, the spy mutants showed significantly reduced enzymatic activities in comparison with the WT protein in planta (~30% in *spy*^*m3*^ triple mutant and 80% in the *spy*^*m2*^ double mutant; Fig. 1g), suggesting that the dimer interface plays an important role for the in vivo function of SPY.

### Structural basis of GDP-fucose recognition by SPY

**Fig. 2:**
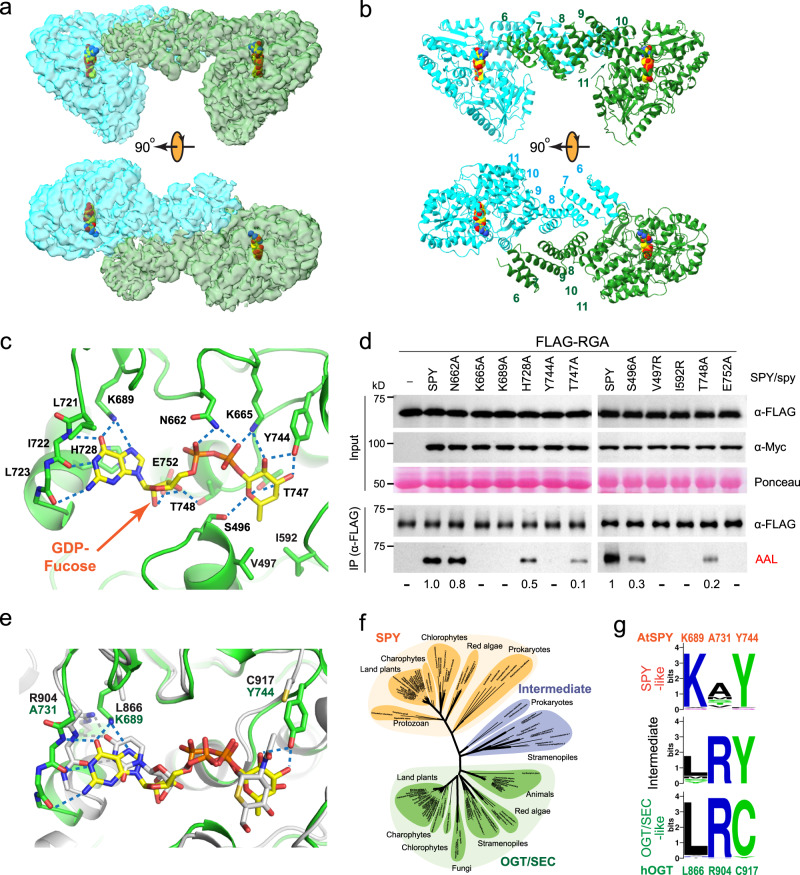
GDP-fucose recognition by SPY. Cryo-EM map and cartoon representation of SPY in complex with GDP-fucose are shown in panels (**a**) and (**b**), respectively. **c** A zoomed-in view of the GDP-fucose recognition by SPY with interfacial residues labeled. Hydrogen bonds are denoted by dashed lines. **d** Effects of active site mutations on the catalytic activity of SPY in planta. FLAG-RGA proteins were expressed alone (−) or co-expressed with Myc-SPY or spy mutant proteins in *N. benthamiana*. Top panel (Input): Immunoblots containing total protein extracts. Bottom panel (α-FLAG IP’ed samples): Immunoblots containing affinity-purified FLAG-RGA proteins. Based on the signals on the AAL blot, the activity of WT SPY was set as 1. –, not detectable. Representative images of three biological repeats (left panel) or two biological repeats (right panel) are shown. Source data are provided as a Source Data file. **e** Differential substrate recognition by SPY and hOGT. Interfacial residues are labeled, and hydrogen bonds are shown as dashed lines. **f** Phylogenetic tree of SPY-Like, OGT/SEC-Like, and intermediate clans of GT41 family enzymes. **g** Signature motifs of three clans.

Despite the overall architectural similarity of SPY and hOGT, these two enzymes have distinct donor substrate specificities: SPY is a dedicated GDP-fucose transferase^3^, whereas the hOGT catalyzes protein *O*-GlcNAcylation using UDP-GlcNAc^27–29^. In order to decipher how SPY selectively recognizes GDP-fucose instead of UDP-GlcNAc, we exploited the knowledge that the SPY catalysis requires the presence of GDP-fucose (donor substrate), the protein substrate (acceptor substrate), and trapped the SPY/GDP-fucose complex by only furnishing GDP-fucose to SPY during cryo*-*EM sample preparation. The cryo*-*EM structure of the SPY/GDP-fucose complex was resolved to ~3.8 Å (Fig. 2a, Supplementary Fig. 4a, b, and Supplementary Table 1). The structure reveals a symmetric SPY dimer (Fig. 2a, b), with each active site containing a fully occupied GDP-fucose that is very well defined by the cryo-EM density (Supplementary Fig. 4b).

The GDP-fucose is recognized by SPY via an elaborate network of interactions (Fig. 2c). The guanosine base is recognized through three hydrogen bonds with the backbone groups of I722 and L723 and two hydrogen bonds with K689. It is additionally sandwiched between the sidechains of L721 and H728 via hydrophobic and π–π stacking interactions, respectively. The nucleoside ribose 2′ and 3′ hydroxyl groups interact with T748 and E752 sidechains through hydrogen bonds. The pyrophosphate group is bridged by N662 and K665. The fucosyl group is held together through hydrogen bonds with sidechains of S496, K665, Y744, and T747, and the methyl group of the fucose ring protrudes into a hydrophobic pocket formed by V497 and I592 sidechains (Fig. 2c). Consistent with our structural observations, alanine substitutions of H728 interacting with the guanosine group, N662 interacting with the pyrophosphate group, and T747 and S496 interacting with the fucose group reduced the catalytic activity of SPY in the transient co*-*expression assays in *N. benthamiana* to 50%, 80%, 10%, and 30%, respectively (Fig. 2d). Alanine substitution of T748 interacting with the nucleoside ribose 2′ hydroxyl group also reduced SPY activity to 20% (Fig. 2d). Strikingly, mutations of K689A, K665A, E752A, Y744A, V497R, and I592R completely abolished the RGA *O-*fucosylation based on immunoblotting with the fucose-specific AAL, highlighting their crucial roles in the recognition of the guanosine, pyrophosphate, ribose 3′ hydroxyl group, and fucose moieties (Fig. 2d). Further corroborating the critical interactions of K665 with the pyrophosphate and fucose groups of GDP-fucose, the spy*-19* (K665M) mutant plant displays very severe phenotypes (early flowering time and much reduced fertility) and completely abolished the POFUT activity in vitro^3,18^.

Several additional spy mutants with mutations in the catalytic domain have been reported^18^. Among them, spy*-18* (ΔM782-S914) shows the most severe phenotype in floral induction and is completely sterile^18^. The large C-terminal deletion in this spy*-18* mutant eliminates the last two helices of the catalytic domain (Supplementary Fig. 5) and likely results in the unfolding of this domain, yielding a null phenotype. Two point mutations in this region, spy*-16/17* (R815W) and spy*-5* (C845Y) confer relatively mild phenotypes^18^, consistent with a limited perturbation of the SPY structure. Four catalytic-domain spy mutants, spy*-12* (G570D), spy*-3* (G593S), spy*-15* (E567K), and spy *13/14* (T572M)^18^, are located near the fucose moiety of the GDP-fucose substrate but do not directly contact fucose. These missense mutations may perturb the local structure and indirectly affect GDP-fucose binding and catalysis. Finally, a frameshift mutation from T880-S914 has been observed in spy*-20*. As this region is absent in the cryo-EM density, the mechanism underlying its genetic phenotype remains to be investigated.

Previous phylogenetic analysis of 53 TPR-containing glycosyltransferases (TPR-GTs) showed that they can be divided into SPY-Like and OGT/SEC-Like clans^16^. Overlay of the cryo-EM structure of the SPY/GDP-fucose complex with the hOGT/UDP-5S-GlcNAc/peptide complex (PDB: 4xif)^30^ shows that in addition to the different protein backbone hydrogen bond patterns between SPY and guanosine base (three hydrogen bonds) and between hOGT and uridine base (two hydrogen bonds), three key residues play an outsized role in defining the glycosylation specificity (Fig. 2e): K689 of SPY favors GDP-fucose by forming two hydrogen bonds with N7 and O6 of the guanosine base, whereas the corresponding residue L866 in hOGT lacks these interactions. Instead, hOGT recognizes the O4 atom of the uridine base by R904 through a single hydrogen bond, whereas this residue is replaced with alanine (A731) in SPY to make room for the K689 sidechain. The long sidechain of K689 may also clash with the uridine ring of UDP-GlcNAc, thus further discriminating against UDP-GlcNAc. Y744 in SPY forms two hydrogen bonds with the fucose (O2 and O3), whereas in GlcNAc, O3 is located on the opposite surface of the hexose ring, and O2 is replaced with the *N*-acetyl group that not only lacks the hydroxyl group but also creates vdW clashes with Y744. On the other hand, such a bulky *N*-acetyl group is well tolerated by the shorter sidechain of C917 in hOGT. Extensive sequence alignment and phylogenetic analysis using 103 TPR-containing glycosyltransferases (TPR-GTs) (Fig. 2f, Supplementary Fig. 6) confirmed that these three key residues are differentially conserved among almost all SPY-Like vs OGT/SEC-Like proteins (Fig. 2g and Supplementary Table 2). Interestingly, in addition to the SPY-Like and OGT /SEC-Like clans, our phylogenetic analysis showed that some of the TPR-GTs from Prokaryotes and Stramenopiles do not cluster with either clan and form an intermediate group (Fig. 2f, Supplementary Fig. 6). Consistent with the phylogenetic tree, proteins within the intermediate group showed mixed patterns for the three key residues (Fig. 2g and Supplementary Table 2).

### The N-terminal TPRs dynamically regulate the conformation of SPY

**Fig. 3:**
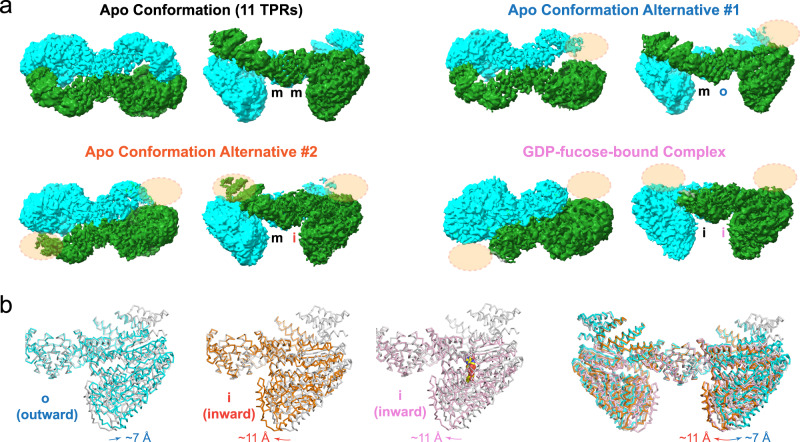
SPY samples multiple conformational states. **a** Cryo-EM maps reveals distinct conformations of SPY in the apo and GDP-fucose-bound states. Missing densities of the TPR region are indicated by peach-colored ovals. The three conformations (outward (“o”), middle (“m”), and inward (“i”)) of the catalytic domain are labeled following their relative position to the central TPRs. **b** Overlay of SPY reveals the movement of the catalytic domain. The apo SPY with 11 TPRs is colored in gray and is used as the reference. Two alternative conformations of apo SPY are colored in blue and brown, respectively, and the GDP-fucose-bound SPY is colored in pale pink.

In addition to the observation of GDP-fucose in the active site, the most noticeable conformational change in the GDP-fucose-bound SPY is the absence of EM densities of the N-terminal TPRs (TPRs 1–5; Fig. 2a, b). Intriguingly, two alternative conformations captured for apo SPY also lack EM densities for the N-terminal TPRs in one or both subunits of the SPY dimer (alternative conformations #1 and #2 in Fig. 3a), suggesting that the N-terminal TPRs of SPY are not stably formed or positioned. Additionally, the catalytic domain shows large displacement in different apo SPY structures when the N-terminal TPRs are absent (Fig. 3a, b).

As TPR1 of one subunit of the symmetric SPY dimer latches onto the catalytic domain of the opposite subunit in the apo state, we reasoned that the presence of the entire 11 TPRs would restrain the mobility of the catalytic domain, which can serve as a reference for conformational comparison. To visualize the motion of the catalytic domain in different conformational states, we compared the three structures (11 TPRs and two alternative conformations) of apo SPY together with that of the SPY/GDP-fucose complex by superimposing their dimeric interface of TPRs 6–9 (residues M218–K359). All four conformations superimposed well in this region with an overall RMSD of <0.8 Å, suggesting that the dimeric interface of SPY remains unchanged despite the movement of the catalytic domain. The apo SPY conformations, in general, lack the C2 symmetry in the absence of full TPRs, suggesting that the two catalytic domains move independently from each other.

Our structural comparison reveals that the SPY catalytic domain can undergo significant inward movement (denoted as the “i” state) toward the center or outward movement (denoted as the “o” state) away from the center in comparison with the catalytic domain of SPY containing the entire 11 TPRs (denoted as the middle or “m” state, Fig. 3a, b). In the protomer A of the alternative conformation #1, the catalytic domain moves further away from the center of the SPY dimer in comparison with the catalytic-domain position of SPY with 11 fully formed TPRs (~7 Å at the tip of the catalytic domain), leaving a very wide gap between the central TPRs and the catalytic domain. In contrast, in the alternative conformation #2, the catalytic domain moves inward to the center of the SPY dimer in comparison with that of the 11 TPR containing SPY (~11 Å at the tip of the catalytic domain), narrowing the space between the catalytic domain and TPRs. Interestingly, particles for the catalytic domain in the inward (“i”) conformation (~270 k) vastly outnumber the particles in the outward (“o”) conformation (~80 k) or in the middle (“m”) conformation (~92 k) containing full 11 TPRs (Supplementary Fig. 1a), suggesting that the inward positioned catalytic domain conformation is likely the more stable state.

The GDP-fucose-bound form of SPY also has the catalytic domain moving toward the center with the same magnitude as in the alternative conformation #2, suggesting that GDP-fucose binding favors the inward conformation pre-existing in the dynamic structural ensemble of apo SPY (Fig. 3b). However, as particles for the GDP-fucose bound SPY complex were selected for the best GDP-fucose density in the active site, we cannot exclude the possibility that GDP-fucose could similarly bind to other conformational states (i.e., “m” and “o” states) observed in apo SPY, but the corresponding particles were excluded from 3D reconstruction due to a less rigid GDP-fucose binding mode in these states resulting in weaker GDP-fucose densities.

### The N-terminal region regulates the substrate binding and catalytic activity of SPY

**Fig. 4:**
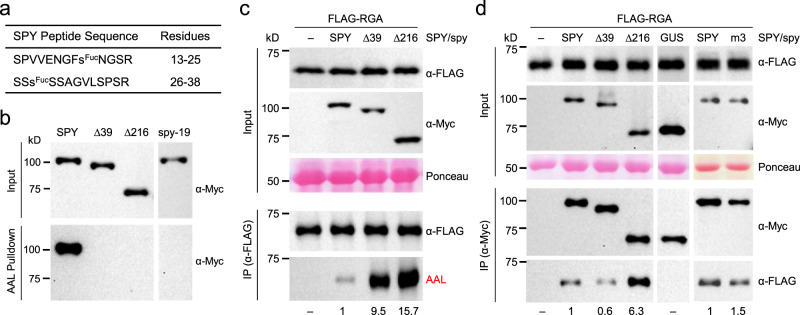
Auto-fucosylation of SPY and the inhibitory role of its N-terminal region on POFUT activity. **a**, **b** SPY was auto-*O*-fucosylated in planta. Myc-SPY or spy mutant proteins were transiently expressed in *N. benthamiana*. Δ39 and Δ216: truncated spy proteins with deletions of 39 and 216 a.a. from the N-terminus. spy-19, a biochemically null mutant with K665M^3^. In (**a**), two *O*-fucosylation sites (S21 and S28) in SPY were identified by ETD-MS/MS analysis (Supplementary Figs. 7, 8). In (**b**), the full-length SPY was auto-*O*-fucosylated, whereas none of the mutant spy proteins was fucosylated. *O*-fucosylated proteins were pulled down by AAL-agarose. Immunoblot containing input (top panel) or AAL-agarose pull-down samples (bottom panel) was probed with an anti-Myc antibody. Representative images of three biological repeats are shown. **c** Deletion of N-terminal peptide (Δ39) and TPRs 1–5 (Δ216) in SPY enhanced its POFUT activity in planta. FLAG-RGA was expressed alone (−) or co-expressed with Myc-SPY or spy in *N. benthamiana*. Top panel (Input): Immunoblots containing total protein extracts. Bottom panel (α-FLAG IP’ed samples): Immunoblots containing affinity-purified FLAG-RGA proteins. Based on the signals on the AAL blot, the activity of WT SPY was set as 1. –, not detectable. **d** Co-IP assays showed that deletion of N-terminal TPRs in SPY (Δ216) significantly increased interaction with RGA in planta, whereas Δ39 or dimerization-deficient spy^m3^ did not. FLAG-RGA was expressed alone (−) or co-expressed with Myc-SPY or spy in *N. benthamiana*. Myc-GUS that does not interact with RGA was included as a negative control^17^. Myc-SPY or spy proteins or Myc-GUS were IP’ed using anti-Myc agarose. Top panel (Input): Immunoblots containing total protein extracts. Bottom panel (α-Myc IP’ed samples): Immunoblots containing IP eluates, and were probed with αMyc or αFLAG as labeled. The relative amounts of FLAG-RGA that were co-IP’ed were calculated by the signals on the αFLAG blot, which were normalized using the IP’ed Myc fusion protein signals on the αMyc blot. FLAG-RGA co-IP’ed by WT SPY was set as 1. –, not detectable. In (**c**) and (**d**), representative images of two biological repeats are shown. Source data for (**b**)–(**d**) are provided as a Source Data file.

Our cryo-EM analysis (Fig. 3) implicates the role of N-terminal TPRs (TPRs 1–5) in regulating the SPY conformation. Unexpectedly, we found that the N-terminal disordered sequence before TPR1 in SPY was auto-*O*-fucosylated when it was transiently expressed in *N. benthamiana*. Two *O*-fucosylation sites, S21 and S28, were identified through electron transfer dissociation-tandem MS (ETD-MS/MS) analysis (Fig. 4a and Supplementary Figs. 7, 8). Deletion of residues 1–39 (Δ39 that removes the disordered N-terminal peptide) or 1–216 (Δ216 that removes both the N-terminal peptide and TPRs 1–5) completely abolished SPY auto-*O*-fucosylation as shown by the AAL pull-down assay, suggesting that the major *O*-fucosylation sites reside within the N-terminal 39-residue peptide of SPY (Fig. 4b). spy-19 (K665M), which lacks POFUT activity^3^, also completely abolished its fucosylation, confirming auto-fucosylation of SPY (Fig. 4b). There was no visible density for this peptide in the cryo-EM maps, indicating that the peptide is likely disordered. Interestingly, we found that the full-length recombinant SPY protein used in our cryo-EM studies purified from insect cells was also fully auto-*O*-fucosylated, as SPY orthologs are absent in animals^16^ (Fig. 2f), and further incubation of purified SPY with GDP-fucose did not increase the fucosylation level of SPY (Supplementary Fig. 9a, b). However, auto-*O*-fucosylation did not prevent the binding of the donor substrate GDP-fucose, as evidenced in our cryo-EM structure of the SPY-GDP-fucose complex (Fig. 2).

To examine the functional roles of this disordered N-terminal 39-residue peptide and TPRs 1–5, we evaluated the effects of their removal in SPY on the POFUT activity toward RGA by the transient co*-*expression assay in *N. benthamiana*. Despite similar expression levels of these spy mutants and WT SPY in planta, there was a striking elevation of the RGA *O-*fucosylation level for both Δ39 and Δ216 over the WT protein: Δ39 increased the POFUT activity over WT SPY by 9.5-fold, and Δ216 mutant activity increased by 15.7-fold (a further ~1.6-fold enhancement from Δ39; Fig. 4c), suggesting that both the N-terminal peptide and TPRs 1–5 of SPY inhibit its POFUT activity. We further examined the RGA interaction with WT SPY and spy mutants by co-IP assays using the transient co*-*expression system in *N. benthamiana*. The Δ39 mutant showed similar RGA binding affinity to that of WT SPY, whereas Δ216 significantly enhanced RGA interaction by 6.3-fold (Fig. 4d). This striking effect of Δ216 (deletion of TPRs 1–5) on RGA binding is unlikely caused by disruption of the SPY dimer formation because the dimerization interface is captured entirely within TPRs 6–9 in our cryo-EM analysis of full-length SPY, and the dimerization-deficient spy^m3^ did not enhance RGA binding (Fig. 4d). Taken together, these results suggest that TPRs 1–5 suppress the SPY catalytic activity and protein substrate binding by restricting the conformational dynamics required for robust substrate binding and catalysis, whereas the N-terminal peptide reduces the POFUT activity of SPY by serving as a competitive inhibitor, likely through inter-SPY dimer inhibition. Consistent with this notion, 2D classes of SPY tetramers formed by side-by-side stacking of SPY dimers can be visualized for the apo protein, in which the N-terminal peptide from one SPY dimer would be positioned close to the catalytic domain of another SPY dimer for *trans* inhibition (Supplementary Fig. 9c).

## Discussion

The extensive sequence similarity between SPY and hOGT has led to the initial misclassification of SPY as an OGT. Through detailed biochemical and genetic characterization, we have revealed SPY as the first nucleocytoplasmic *O*-fucosyltransferase (POFUT). Our cryo-EM analysis reveals three distinct sets of residues that define the differential specificity for the nucleotide (recognition of guanosine by K689/A731 in SPY and uridine by L866/R904 in hOGT) and the attached sugar moiety (recognition of fucose by Y744 in SPY and GlcNAc by C917 in hOGT). Such structural observations provide a molecular basis to rationalize the phylogenetic tree of the GT41 family of TPR domain-containing glycosyltransferases and key signatures (Fig. 2f, g).

Although our cryo-EM structures of SPY and its complex with GDP-fucose do not contain the peptide substrate, the extensive sequence and structural homology of SPY with hOGT have made it possible to speculate on the catalytic mechanism and build a model of SPY/GDP-fucose/peptide complex by superimposing the catalytic domain of existing coordinates of hOGT complexes, such as the hOGT/UDP-5S-GlcNAc/substrate peptide complex (PDB: 4xif)^30^. In the superimposed complex (Supplementary Fig. 10), the UDP-GlcNAc molecule overlays well with GDP-fucose, and the substrate serine residue poised for the nucleophilic attack of the C1 carbon of GlcNAc is similarly positioned for attacking the C1 carbon of GDP-fucose, suggesting that SPY likely undergoes an SN2 reaction similar to hOGT^25,31^, POFUT1/2^19,32,33^, and FUT8^34^. Whether the substrate serine residue requires activation by a catalytic general base from SPY or by the phosphate group from GDP-fucose requires further investigation. Furthermore, through a rigid body rotation of the Tab1 peptide in the hOGT complex (PDB: 5lvv)^35^ to avoid vdW clashes with supercoiled TPRs in SPY, we are able to model the SPY N-terminal peptide in an extended conformation based on the backbone of the Tab1 peptide and identify potential interacting Asn ladder^36^ residues (N333, N334, N367, and N368) lining the TPRs for substrate interaction (Supplementary Fig. 11).

Our cryo-EM analysis has painted a surprisingly dynamic picture of SPY, with its catalytic domain moving along the central and C-terminal TPRs over a distance as large as 18 Å from the outward state to the inward state (Fig. 3 and Supplementary Fig. 11). Such a movement of the catalytic domain is coupled with the collapse of the N-terminal TPRs (TPRs 1–5), which, when becoming fully structured, would hold the catalytic domain in a rigid conformation (middle state; Fig. 3 and Supplementary Fig. 11). Deletion of the N-terminal TPRs 1–5 (Δ216) significantly elevated the SPY interaction with RGA by ~6.3-fold (Fig. 4d), suggesting that the rigid conformation with 11 TPRs locking the catalytic domain may not be optimal for the SPY-RGA interaction and catalysis. Furthermore, as the donor substrate GDP-fucose is largely recognized by the catalytic domain alone, whereas the recipient substrate peptide is tethered by the Asn ladder within the TPRs, movement of the catalytic domain with the bound GDP-fucose may dynamically position GDP-fucose close to the receptor serine/threonine residues from diverse substrate sequences^37,38^ in the active site for efficient catalysis despite the local variation of the substrate binding mode caused by the different peptide sequences.

SPY*-Like* genes are highly conserved in plants, algae, protists, and bacteria. However, very few of these SPY*-Like* genes and their encoded proteins have been characterized. Our structural and functional study of SPY defines the critical residues for POFUT activity in SPY-like proteins, indicating that *O*-fucosylation of intracellular proteins is a conserved regulatory mechanism in diverse organisms.

While this manuscript was under revision, a crystal structure of auto-inhibited *Arabidopsis* SPY in complex with GDP was reported^39^. The superimposition of the crystal structure with the cryo-EM structure shows an overall excellent agreement of the structural features. Interestingly, although Zhu et al. concluded that it is unlikely for SPY TPRs to display any conformational flexibility^39^, our cryo-EM analysis readily reveals the presence of multiple conformations of the TPRs and catalytic domain orientation (Fig. 3). Comparison of the crystal structure and cryo-EM structures shows that the SPY catalytic domain of the crystal structure is located between the middle and inward states of the cryo-EM structures, likely reflecting a conformational averaging in the crystal lattice of these two solution states (Supplementary Fig. 12).

Superimposition of the catalytic domain of the crystal structure of the SPY/GDP complex with that of the cryo-EM structure of the SPY/GDP-fucose complex reveals a consistent binding pose of the GDP moiety between the crystal and cryo-EM structures and that the receptor serine sidechain hydroxyl group from the auto-inhibitory N-terminal SPY peptide is located in proximity to the C1 carbon of the GDP-fucose ring (Supplementary Fig. 13), lending further support to the proposed mechanism of a substrate-assisted SN2 reaction proposed here and by Zhu et al.^39^. As recent proteomic analysis of SPY substrates did not reveal a distinct pattern around the *O*-fucosylation sites, except the enrichment of serine residues^37,38^, how SPY selectively recognizes diverse peptide sequences requires further investigation. Intriguingly, although our truncation study also suggested a model of self-inhibition from the N-terminal disordered peptide (residues 1–39) in SPY, we did not observe any evidence of the T-shaped *trans* inhibitory state within a dimer of SPY dimer as observed in the crystal structure. Instead, we observed a cryo-EM 2D class of side-by-side stacked dimer of SPY from flash-frozen solution state (Supplementary Fig. 9), which would be similarly compatible with a *trans* N-terminal peptide inhibition, but with twice the binding interface, suggesting that the T-shaped SPY tetramer might be stabilized by the crystal lattice environment.

Another unique insight from our cryo-EM analysis is that the N-terminal TPRs of SPY may play a regulatory role in modulating SPY activity. Removal of the N-terminal TPRs (TPRs 1–5) not only further elevated the catalytic activity of SPYΔ39 devoid of the N-terminal inhibitor peptide but also significantly elevated its binding affinity toward the full-length RGA substrate in planta. How the N-terminal TPRs regulate the protein substrate binding of the full-length SPY requires further investigation. It is possible that TPRs 1–5 of SPY might directly or indirectly interfere with SPY-RGA interaction. Alternatively, TPRs 1–5 (either in the folded or unfolded conformations) might interact with other protein substrate(s) and/or regulatory protein(s), which may directly or indirectly regulate SPY activity by blocking the SPY-RGA interaction in planta. Future quantitative measurements of SPY toward recombinant and functionally validated full-length RGA will help resolve whether the SPY N-terminal TPRs directly modulate the substrate binding.

## Methods

### Cloning and expression of full-length *Arabidopsis thaliana* SPY

The SPY-WT-TEV-His_10_-Strep fusion construct was generated using the in-fusion cloning strategy. The full-length *Arabidopsis thaliana* SPY gene was amplified using FP1 and RP1 primers, and the purified insert was used as a template for the second round of PCR amplification using FP1 and RP2 primers (Supplementary Table 3). The insert was purified and cloned into the pFASTBac1 vector at *Eco*R1 and *Hin*dIII sites. Recombinant Baculovirus for SPY expression was generated and amplified following the manufacturer’s protocol (Thermo Fisher Scientific) using Sf9 cells (Expression Systems). For protein expression*, Trichoplusia ni* (High Five^TM^) cells were cultured in the ESF 921 insect cell culture media (Expression Systems) at 27 °C; the suspension cell culture was infected with a high titer baculovirus stock and harvested after 56 h by centrifugation (200×*g* for 5 min). Cell pellets were resuspended and lysed by sonication in the purification buffer (25 mM HEPES pH 7.5 and 150 mM NaCl) supplemented with protease inhibitors (15 µM leupeptin, 1 µM pepstatin A, 2 µM E-64, 0.1 µM aprotinin, 1 mM phenylmethylsulphonyl fluoride), 1 mM β-ME. Following centrifugation at 15,000 rpm at 4 °C for 15 min, the cell lysate was subjected to tandem affinity purification using Talon metal affinity resin (Takara Bio USA, Inc.) followed by Strep-Tactin Superflow Plus resin (Qiagen). The eluates were analyzed on SDS-PAGE gel, concentrated, and further purified by size-exclusion chromatography using the Superose® 6 increase 10/300 GL column (Cytiva) pre-equilibrated with buffer containing 25 mM HEPES (pH 7.5) and 150 mM NaCl, 2 mM DTT. The peak fractions were concentrated to 0.8 mg/mL for cryo-EM grid preparation. For the SPY-GDP-fucose complex, 10 mM of GDP-L-fucose (Biosynth International Inc.) dissolved in 25 mM HEPES pH 7.5, 150 mM NaCl was added to apo SPY-WT prior to cryo-EM grid preparation.

SPY dimer interface mutants (spy^m2^ and spy^m3^) were generated from the WT SPY construct as the template via overlapping PCR using L320A_F324A_FP, L320A_F324A_RP, W292A_FP, and W292A_RP primers (Supplementary Table 3) followed by in-fusion cloning. The mutant constructs were verified by DNA sequencing, expressed, and purified similarly to the wild-type SPY protein.

### Cryo-EM sample preparation and data collection

The cryo-EM grids were prepared using the Leica EM GP2 Automatic Plunge Freezer at 16 °C and 95% humidity. The *C-flat R1.2/1.3 300-mesh* grids *(Protochips, Inc.)* were glow-discharged using the Tergeo-EM plasma cleaner (Pie Scientific LLC). Then, 3 µL samples of SPY in the absence of GDP-L-fucose (apo SPY) or in the presence of 10 mM GDP-L-fucose (~0.8 mg/mL; SPY/GDP-fucose complex) were applied to the grids and blotted for 2–3 s with Whatman # 1 filter paper (Whatman International Ltd.) to remove excess sample and plunge-frozen in liquid ethane cooled by liquid nitrogen.

For the apo SPY sample, a total of 10,000 movies were recorded on FEI Talos Arctica electron microscope (Thermo Fisher Scientific) operated at 200 kV equipped with a K3 direct electron detector (Gatan, Inc.) operated in the counting mode using SerialEM (version 3.8.7)^40^. Movies were collected at a nominal magnification of ×54,900 using a pixel size of 0.88 Å/pix with a defocus range from −2.0 to −0.5 μm. Each stack was exposed for 2.7 s with an exposure time of 0.045 s per frame. The total dose was approximately 55 e^−^/Å^2^ distributed over 60 frames.

For the SPY/GDP-fucose complex, a total of 4988 movies were recorded on FEI Titan Krios electron microscope (Thermo Fisher Scientific) operated at 300 kV equipped with a K3 direct electron detector (Gatan, Inc.) operated in the counting mode. Movies were collected at a nominal magnification of ×81,000 using a pixel size of 1.08 Å/pix with a defocus range from −2.0 to −0.8 μm using Latitude^TM^ S (Version 3.51.3719.0, Gatan, Inc.) automated image acquisition package. Each stack was exposed for 4.62 s with an exposure time of 0.077 s per frame, resulting in 60 frames per stack. The total dose was approximately 52.4 e^−^/Å^2^ for each stack.

### Cryo-EM data processing and model building

Data processing for the SPY dataset is summarized in Supplementary Fig. 1. For the apo SPY dataset, movie alignment and contrast transfer function (CTF) estimation were performed with the patch motion correction model and patch CTF estimation module in cryoSPARC^41^. Then, 8748 micrographs were selected from a total of 10,000 images based on the CTF fitting resolution using a cutoff value of 4.0 Å. A total of ~8M particles were selected using templates generated from the 3D EM map of the SPY/GDP-fucose complex (below). After multiple rounds of 2D classification, a total of 654,539 particles were selected for ab initio reconstruction and heterogeneous refinement containing three classes. Two classes with high-resolution features beyond 4 Å were selected for further analysis. The class with the least features of the TPRs was subject to homogeneous refinement and nonuniform refinement, yielding a final cryo-EM map at 3.6 Å resolution. The other class with more complete TPR features was subject to 3D classification, and two representative classes featuring the complete TPR features on both protomers or one protomer were selected for further homogeneous and nonuniform refinement under C2 and C1 symmetry restraints, yielding final reconstructed cryo-EM maps at 3.7 Å and 3.9 Å, respectively.

Data processing for the SPY/GDP-Fucose dataset is summarized in Supplementary Fig. 4. Briefly, movie alignment was done with the patch motion correction module in cryoSPARC^41^, and the parameters of the contrast transfer function were determined on the motion-corrected sum of frames using CTFFIND4.1^42^. Then, 3982 micrographs were selected from a total of 4988 images based on the CTF fitting resolution using a cutoff of 4.5 Å. A total of ~4M particles were boxed out using template-free particle picking and extracted with a binning factor of 2. Two consecutive rounds of 2D classification were performed to clean the extracted particles. A total of 497,517 clean particles were used for further processing. The 2D class averages showed the projections from both SPY dimers and monomers. With that knowledge, three classes were generated using an ab initio reconstruction job in cryoSPARC, followed by one round of heterogeneous refinement to separate the particles based on the three initial reconstructions. Approximately 215k particles belonging to SPY monomers were used for homogeneous refinement yielding a ~7 Å map. Considering the small size and asymmetric shape of the monomer, no further processing was conducted. A subset of 282k particles showing the SPY dimer features was selected and re-extracted using a box size of 320 pixels and binning 1 to perform another round of heterogeneous refinement. The class with better resolution and features was selected and subjected to homogeneous refinement without applying symmetry. Nonuniform refinement was then performed using C2 symmetry which yielded a reconstruction with an overall resolution of 3.8 Å. To improve the catalytic domain of SPY, focus masks covering each protomer were used for focused local refinement after symmetry expansion resulting in 3.1 Å maps for both sides. An overall composite SPY/GDP map was generated from the two locally refined maps and the overall map using Phenix^43^.

The molecular models of SPY and the SPY/GDP-fucose complex were constructed by fitting secondary structures of the AlphaFold^44^ model of the SPY monomer (https://alphafold.ebi.ac.uk/entry/Q96301) into the cryo-EM densities and further refined through iterative editing in COOT^45^ and refinement in Phenix^43^.

### Plasmid construction for in planta studies and for producing recombinant protein in *Escherichia coli*

pEarleyGate203, pEG203-SPY (35S:Myc-SPY), pEG203-3TPR-SPY (35S:Myc-3TPR-SPY = Δ325), pEG203-spy-19 (35S:Myc-spy-19), pEG203-SEC (35S:Myc-SEC), pEG100-3xFR (35S:FLAG-RGA), and pTrc-His-MBP-3TPR-SPY (for expressing 3TPR-SPY in *E. coli*) have been previously described^3,17,46^. Primers for plasmid construction are listed in Supplementary Table 3. Plasmids used in this study were generated using standard molecular biology techniques, as summarized in Supplementary Table 4. All DNA fragments generated by PCR amplification were sequenced to ensure that no mutations were introduced.

### In vitro POFUT assay

To detect auto-fucosylation, the purified recombinant SPY from insect cells was used in the in vitro enzyme assays as described previously^3^, except that no additional protein substrates were included.

### Transient expression of FLAG-RGA and Myc-SPY/spy in *Nicotiana benthamiana*

Agro-infiltrations were performed using *Agrobacterium tumefaciens* strain GV3101 pMP90 carrying different constructs and 3-week-old plants of *N. benthamiana* as described previously^17,47^. For co-expression experiments, *Agrobacterium* culture containing individual constructs was combined before infiltration. In each set of assays, the amount of individual culture was adjusted to ensure similar levels of SPY and spy mutant proteins were present among the samples. Proteins were extracted 2 days after agroinfiltration for protein gel/blot analyses.

### Tandem affinity purification of His-FLAG-RGA proteins from *N. benthamiana*

The FLAG-RGA protein (containing a 6xHis-3xFLAG-tag) was transiently expressed in tobacco and tandem affinity-purified using a His-Bind resin followed by monoclonal anti-FLAG-mouse M2 antibody-agarose beads (Sigma-Aldrich, A2220) as described^3,17^, except at a smaller scale with 0.5 g of starting tissue. All purified proteins were quantified against a protein standard by PAGE followed by Oriole gel staining (Bio-Rad), and by anti-FLAG immunoblot analysis.

### Protein blot analyses

To detect and quantify FLAG-RGA proteins, total protein extracts or affinity-purified proteins from *N. benthamiana* were analyzed by immunoblot analysis using an anti-FLAG mouse monoclonal M2 antibody conjugated with horseradish peroxidase (HRP) (Sigma-Aldrich, A8592; 10,000× dilution), following procedures described previously^3^. An anti-cMyc rabbit polyclonal antibody conjugated with HRP (Sigma-Aldrich, A5598; 5000× dilution) was used to detect Myc-SPY. To quantify *O*-fucosylation levels in FLAG-RGA, AAL blot analysis was performed using 80 ng purified FLAG-RGA proteins from each *N. benthamiana* tissue. The nitrocellulose membrane was blocked with 3% bovine serum albumin (BSA) in TBST-500 (20 mM Tris pH 7.5, 500 mM NaCl, 0.05% Tween-20) for 2 h at room temperature (RT), followed by 1-h incubation with 30,000× dilution of biotinylated-*Aleuria aurantia* lectin (AAL-biotin, Vector Labs, B-1395, 1 mg/mL) in TBST-500 and 3% BSA. After washing with TBST-500, the blot was incubated for 30 min at RT with Streptavidin-HRP (100,000× dilution, Jackson Immunoresearch Labs, 016-030-084) in TBST-500 and 3% BSA. SuperSignal Pico chemiluminescent reagent and an iBright Imaging System (Thermo Fisher Scientific) were used to detect and quantify the HRP signals.

### AAL-agarose pull-down and co-IP assays

AAL-agarose pull-down assays were performed to examine the levels of *O*-fucosylation in WT SPY and spy mutants. For the pull-down assays, Myc-SPY and Myc-spy protein amounts in different *N. benthamiana* tissues were adjusted to be the same by adding non-infiltrated leaf tissues. Then, 250 mg of agro-infiltrated leaf tissue was homogenized in 1.5 mL of Extraction Buffer (50 mM Tris-HCl, pH 7.5, 500 mM NaCl, 0.5% Triton X-100, 2.5 mM 2-mercaptoethanol, 20 µM MG-132, 1× Complete EDTA-free protease inhibitors (Sigma-Aldrich) on ice and centrifuged at 15,000×*g* for 10 min at 4 °C. After this, 20 µL of each protein extract was mixed with 30 µL of 2× Laemmli Sample Buffer (LSB) (Bio-Rad) for input analysis. To 1.5 mL of extract, 20 µL of AAL-agarose beads (Vector Labs, AL-1393-2, 2 mg lectin/mL) were added and incubated with rotation for 1.5 h at 4 °C. The beads were washed with extraction buffer, four times with TBST-500, and once with 20 mM Tris-HCl, pH 7.5, and were boiled in 50 µL of 2 × LSB for gel blot analysis.

To examine the differential binding affinity of SPY and spy mutants to RGA, FLAG-RGA was expressed alone or co-expressed with Myc-SPY or Myc-spy mutant proteins in *N. benthamiana*, and subsequent co-IP assays using anti-cMyc rabbit antibody conjugated agarose beads (Sigma-Aldrich, A7470) were performed as described^17^.

### Sequence alignment and phylogenetic analysis

To search for SPY and SEC homologs across the tree of life, we downloaded proteomes of representative species from PhycoCosm^48^ and Phytozome^49^ databases and used Orthofinder (v2.5.4)^50^ to cluster the protein sequences into orthogroups. Three orthogroups contained SPY and SEC-like sequences and were combined, along with the OGT sequences compiled by Olszewski et al.^16^. We aligned the protein sequences using MAFFT v7^51^ followed by quality trimming with trimAl (v1.4)^52^. The trimmed alignment containing only sequences of the C-terminal domain was used for the phylogenetic analysis. Maximum likelihood phylogenetic reconstruction was done by IQ-TREE (v2.0.3)^53^ with automatic model selection and rapid bootstrapping (1000 replicates) to assess branch support. The resulting tree was plotted by iTOL (v6)^54^.

### Enrichment of *O*-fucosylated SPY peptides for MS analyses

*Nicotiana benthamiana* leaves were agro-infiltrated with 35S:Myc-SPY *Agrobacterium* strain^17^. To enrich for *O*-fucosylated proteins prior to trypsin digestion, a modified procedure combining two published methods^8,55^ was used. Starting with 1 g of 35S:Myc-SPY agro-infiltrated leaf tissue, a pellet of total protein after phenol-extraction and precipitation was obtained, according to Xu et al.^55^. The AAL pull-down procedure of Bandini et al.^8^ was then followed with some modifications. The protein pellet was dissolved in 500 µL of solubilization buffer (40 mM Tris-HCl, pH 7.5, 150 mM NaCl, 0.1 mM DTT), incubated at 50 °C for 10 min, and centrifuged at top speed for 15 min at RT. The soluble fraction was recovered. A 200-μL aliquot was diluted with 13.5 mL of Protein Dilution buffer (PD; 20 mM Tris-HCl, pH 7.5, 150 mM NaCl, 0.8% N-octyl-glucopyranoside (w/v), 40 mM DTT, EDTA-free SigmaFast protease inhibitors) in a 15-mL conical tube. To pull down fucosylated proteins, 30 µg of biotinylated AAL was added to the protein dilution and incubated with rotation for 2 h at 4 °C. Then, 500 µg of prewashed magnetic Streptavidin beads were mixed in and continued incubation with rotation for 30 min, and the beads were then collected with a magnetic rack and washed five times with 1 mL PDS buffer (PD buffer + 0.03% SDS). Fucosylated proteins were eluted from beads with 100 μL PDS buffer and 0.2 M αMeFuc, for 16 h with mixing at 4 °C, followed by a second elution with 70 μL of the same buffer for 2 h. Both eluates were combined, and proteins precipitated with 8 volumes of 0.1 M ammonium acetate in MeOH at −20 °C for 16 h. The washed pellet was dissolved in 100 µL of 50 mM ammonium bicarbonate, pH 8, for trypsin digestion as described above, at a 1:100 (w/w) ratio.

### Identification of *O*-fucosylation sites in SPY by liquid chromatography (LC)-electron transfer dissociation (ETD), collision-activated dissociation (CAD), and higher-energy collisional dissociation (HCD)-tandem MS (MS/MS) analyses

Trypsin-digested proteins from *N. benthamiana* (expressing *Arabidopsis* SPY) were separated by HPLC and analyzed on a Thermo™ Orbitrap Fusion™ Tribrid™ mass spectrometer equipped with ETD^56^. HPLC was performed as described previously^3^. MS1 spectra were acquired in the Orbitrap with a resolution of 120,000, followed by a data-dependent, 3-s TopN method with a neutral loss trigger. Precursors were isolated by resolving quadrupole with a 3 m/z window. CAD MS2 spectra were collected in the Orbitrap with a resolution of 15,000. A neutral loss trigger selected precursors exhibiting the characteristic loss of one to three fucose units in the Orbitrap CAD MS2 spectra for additional fragmentation events. An HCD MS2 (25% NCE) was collected in the Orbitrap with a resolution of 15,000. CAD MS2, CAD MS3, and ETD MS2 spectra were acquired in the ion trap at a normal scan rate. Calibrated reaction times were used for ETD events. Dynamic exclusion was enabled with a repeat count of 2 and an exclusion duration of 6 s.

Data files were searched using Byonic version 3.8.13 (Protein Metrics) (Bern et al.). Data files were searched against a database containing the sequence of SPY (N-terminally Myc-tagged SPY, Uniprot Accession Q96301) and the UniprotKB/Swiss-Prot protein sequence database for *N. benthamiana*. Search parameters included specific cleavage C-terminal to R and K residues with up to five allowed missed cleavages, 10 ppm tolerance for precursor mass, 15 ppm mass tolerance for high-resolution MS2s, and 0.35 Da mass tolerance for low-resolution MS2s. Variable modifications selected included oxidation of Met residues, phosphorylation of Ser, Thr, and Tyr residues, alkylation of Cys residues, and O-GlcNAcylation, O-fucosylation, and O-hexosylation of Ser and Thr residues. No protein false discovery rate cutoff or score cutoff was applied prior to the output of search results. Peptide sequences were validated by manual interpretation of MS2 data. Modification sites were determined manually based on ETD MS2 spectra.

### Reporting summary

Further information on research design is available in the Nature Portfolio Reporting Summary linked to this article.

## Supplementary information

Supplementary Information

Reporting Summary

## Data Availability

The data that supports this study are available from the corresponding authors upon request. The cryo-EM structures of apo SPY in three conformations and the SPY/GDP-fucose complex have been deposited to the Protein Data Bank (www.pdb.org) with access codes 8DTF, 8DTG, 8DTH, and 8DTI. The three corresponding maps of apo SPY and the overall and composite maps of the SPY/GDP-fucose complex have been deposited to EMDB under the access codes EMD-27696, EMD-27697, EMD-27698, EMD-27700, and EMD-27699 respectively. The mass spectrometry proteomics data have been deposited to the ProteomeXchange Consortium via the PRIDE^57^ partner repository with the dataset identifier PXD040480. Source data are provided with this paper.

## Source data

Source Data
