# Supplementary material for: Structure and dynamics of the Arabidopsis O-fucosyltransferase SPINDLY
Source: Nat Commun. 2023 Mar 20;14:1538. doi: 10.1038/s41467-023-37279-1 (PMC10027727; doi:10.1038/s41467-023-37279-1)
Supplement: Supplementary file 1 — Supplementary Information [file 41467_2023_37279_MOESM1_ESM.pdf]

SUPPLEMENTARY INFORMATION

Structure and dynamics of the *Arabidopsis O*-fucosyltransferase SPINDLY

Shivesh Kumar[1†], Yan Wang[2†], Ye Zhou[3†], Lucas Dillard[4], Fay-Wei Li[5,6], Carly A. Sciandra[1], Ning Sui[2], Rodolfo Zentella[2], Emily Zahn[7], Jeffrey Shabanowitz[7], Donald F. Hunt[7,8], Mario J. Borgnia[4], Alberto Bartesaghi[1,3,9*], Tai-ping Sun[2*] and Pei Zhou[1*]

[1]Department of Biochemistry, Duke University School of Medicine, Durham, NC 27710

[2]Department of Biology, Duke University, Durham, NC 27708

[3]Department of Computer Science, Duke University, Durham, NC 27705

[4]Genome Integrity and Structural Biology Laboratory, National Institute of Environmental Health Sciences, Research Triangle Park, NC 27709

[5]Plant Biology Section, Cornell University, Ithaca, NY 14853

[6]Boyce Thompson Institute, Ithaca, NY 14853

[7]Department of Chemistry, University of Virginia, Charlottesville, VA 22904

[8]Department of Pathology, University of Virginia, Charlottesville, VA 22903

[9]Department of Electrical and Computer Engineering, Duke University, Durham, NC 27708

[†] These authors contributed equally.

*Correspondence should be addressed to: peizhou@biochem.duke.edu; tps@duke.edu; alberto.bartesaghi@duke.edu

SUPPLEMENTARY FIGURES

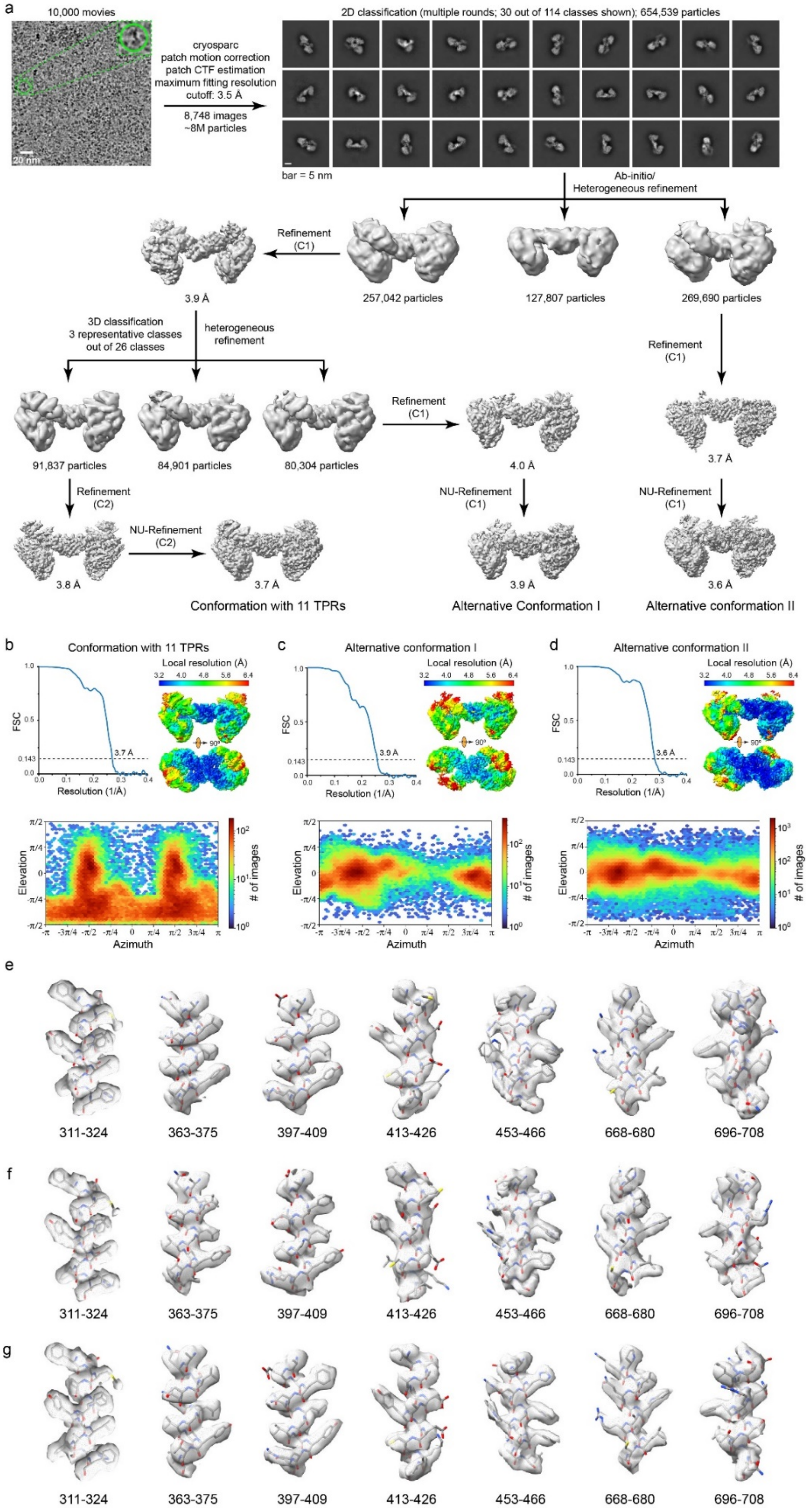

Supplementary Figure 1. Cryo-EM reconstruction of apo SPY. a, Flowchart of the reconstruction. Details are described in the Methods. The global Fourier Shell Correlation (FSC) curves, local resolution maps, and particle distributions for conformation with 11 TPRs, alternative conformation 1, and alternative conformation 2 are shown in panels **b**, **c**, and **d,** respectively. Representative regions of the EM density maps for conformation with 11 TPRs, alternative conformation 1, and alternative conformation 2 are shown in panels **e**, **f**, and **g,** respectively

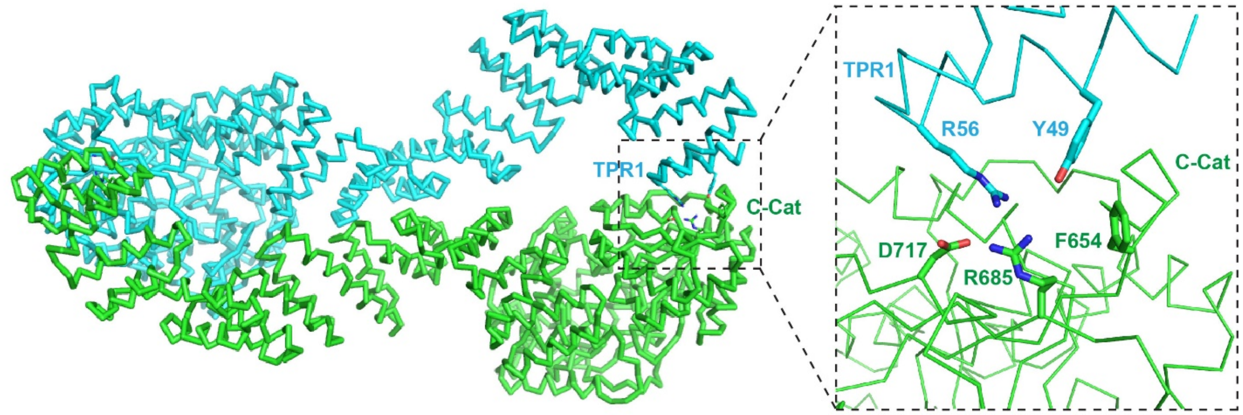

Supplementary Figure 2. TPR1 of SPY interacts with the C-terminal half of the catalytic domain (C-Cat). Interfacial residues are shown in the stick model and labeled.

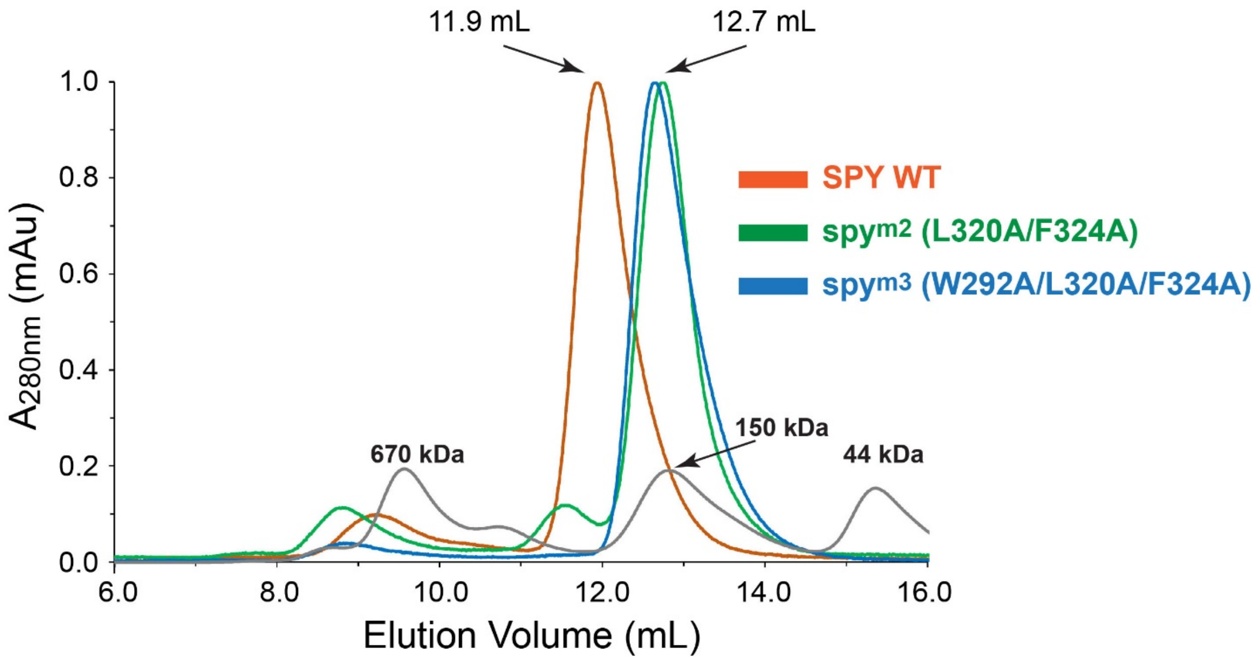

Supplementary Figure 3. Size-exclusion chromatography profiles of WT SPY and spy^{m2} (L320A/F324A) and spy^{m3} (W292A/L320A/F324A) mutants. The chromatography runs were performed using the Superdex 200 increase 10/30 GL column. FPLC traces for WT SPY, spy^{m3}, spy^{m3}, and protein markers are shown in brown, green, blue, and grey, respectively. The size of the protein markers are labeled.

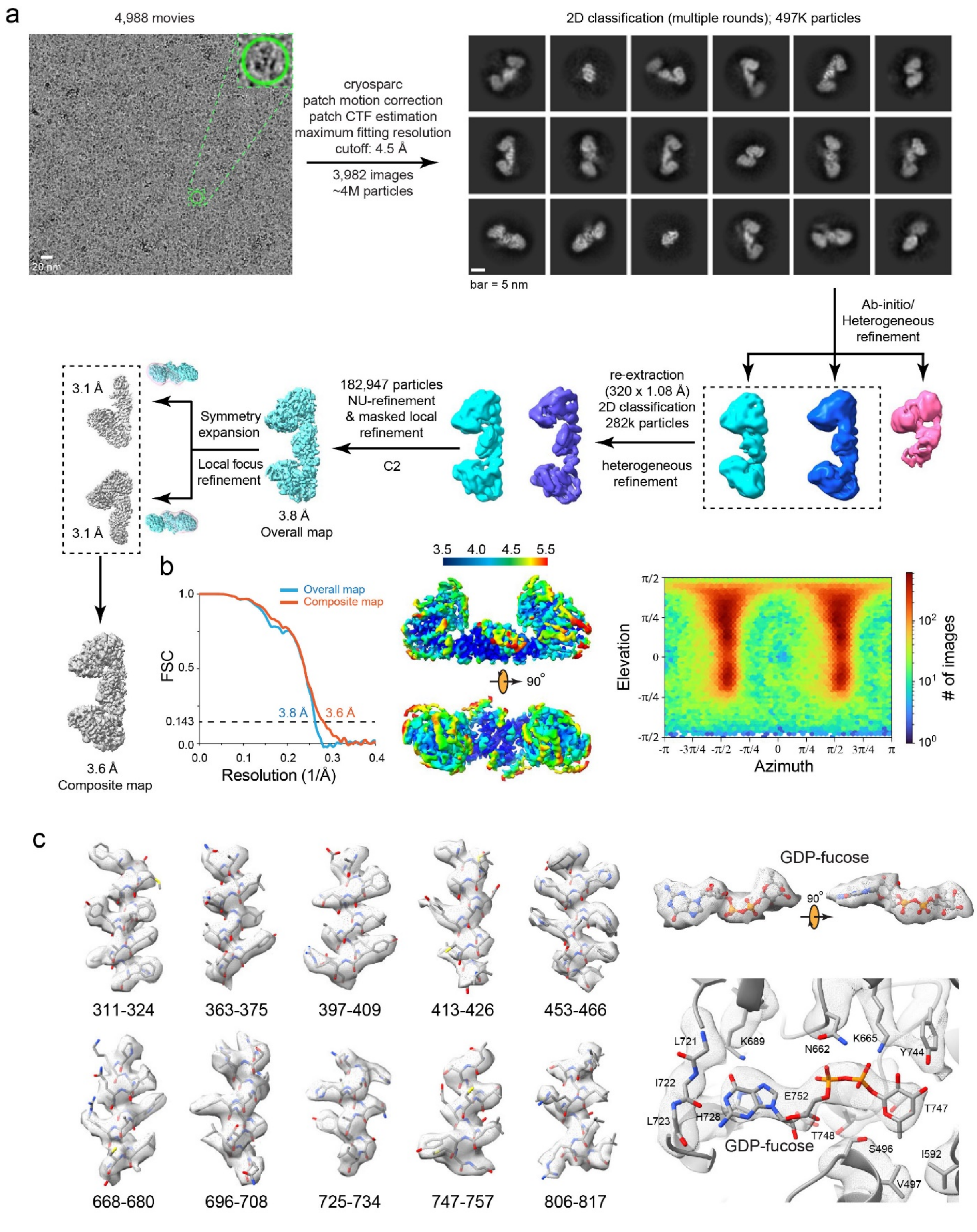

Supplementary Figure 4. Cryo-EM reconstruction of SPY in complex with GDP-fucose. a, Flowchart of the reconstruction. Details are described in the Methods. **b**, The global Fourier Shell Correlation (FSC) curves, local resolution maps, and particle distributions. **c**, Representative regions of the EM density maps.

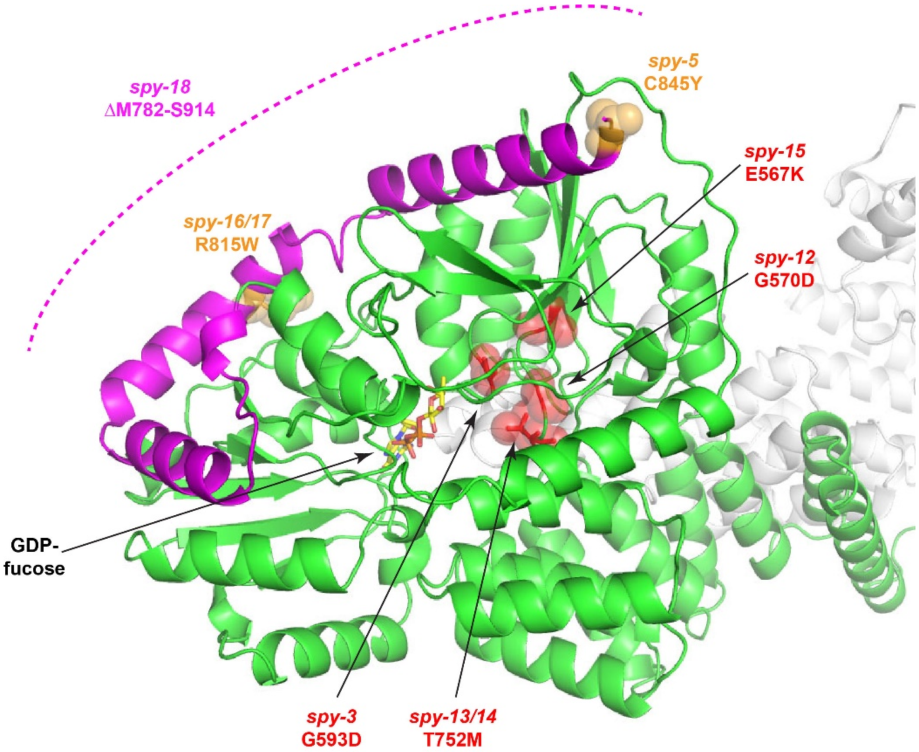

Supplementary Figure 5. Mapping of *spy* genetic mutants in the catalytic domain onto the cryo-EM structure. SPY is shown in the cartoon diagram, with the two monomeric subunits colored in green and grey. Residues deleted in *spy-18* (ΔM782-S914) are colored in purple. Point mutations are shown in the sphere model with the sidechains shown in the stick model. Mutates residues in the C-terminal helices and near the fucose moiety are colored in orange and red, respectively.

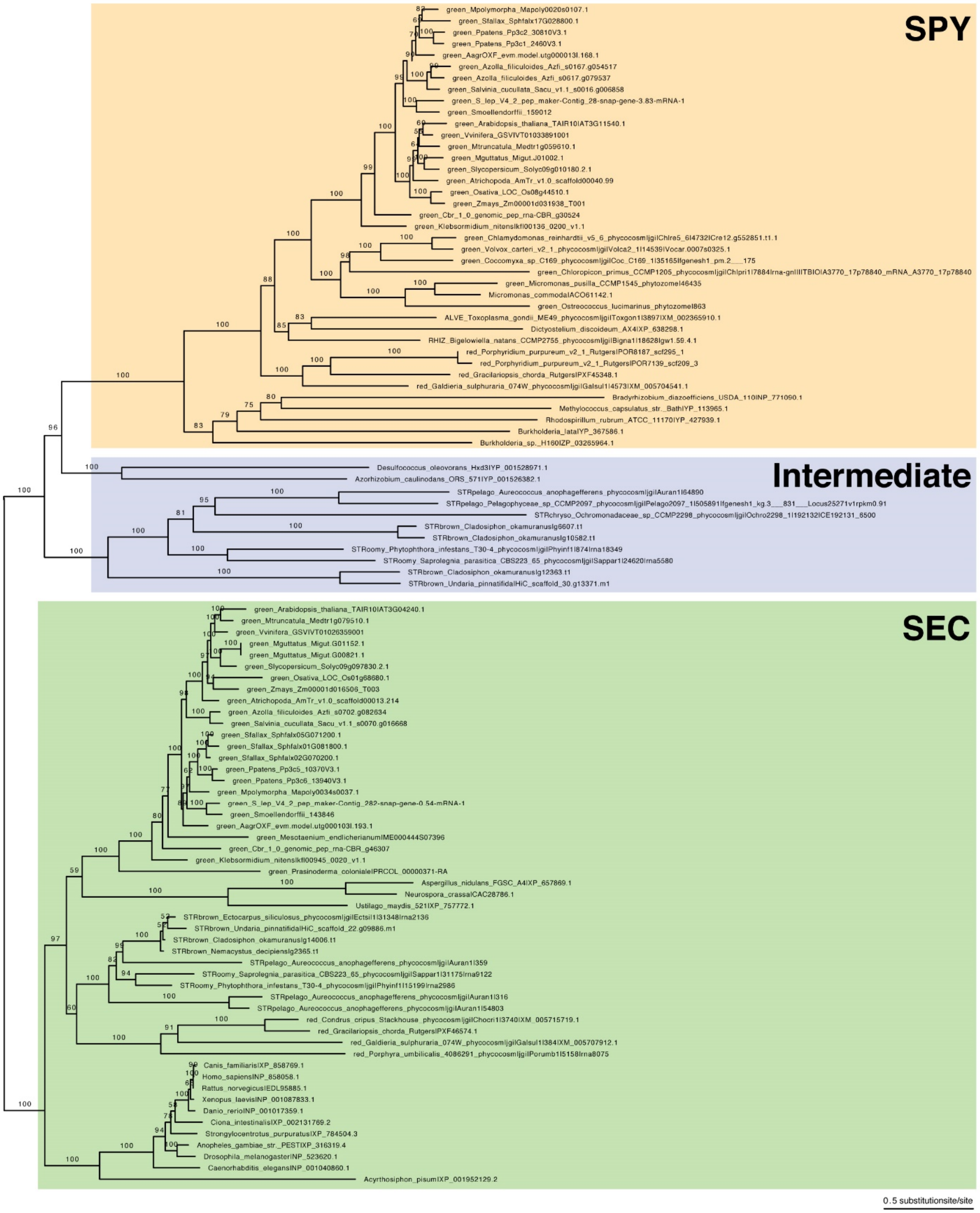

Supplementary Figure 6. Phylogenetic tree of TPR-containing GT41 family proteins. Three distinct clans (SPY-Like, OGT/SEC-Like, and intermediate) are highlighted in brown, pale blue, and green separately. Numbers above branches are bootstrap support values.

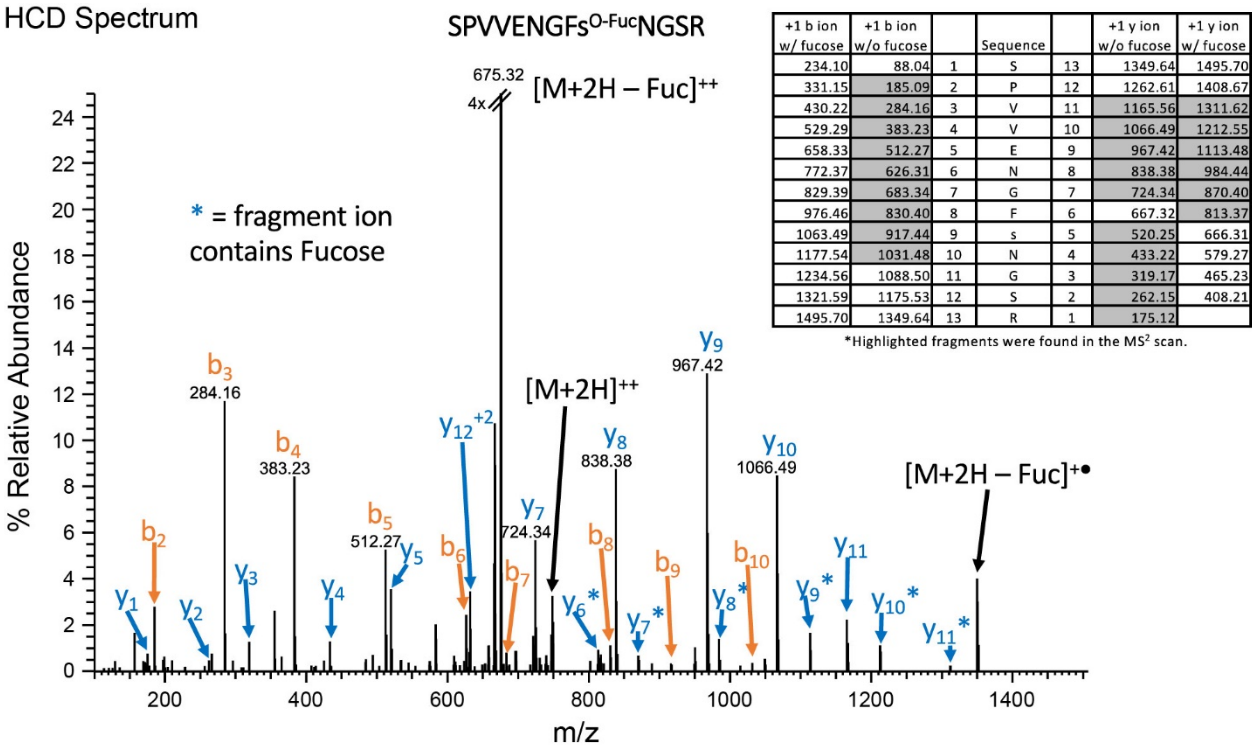

+1 b ion w/ fucose	+1 b ion w/o fucose		Sequence		+1 y ion w/o fucose	+1 y ion w/ fucose
234.10	88.04	1	S	13	1349.64	1495.70
331.15	185.09	2	P	12	1262.61	1408.67
430.22	284.16	3	V	11	1165.56	1311.62
529.29	383.23	4	V	10	1066.49	1212.55
658.33	512.27	5	E	9	967.42	1113.48
772.37	626.31	6	N	8	838.38	984.44
829.39	683.34	7	G	7	724.34	870.40
976.46	830.40	8	F	6	667.32	813.37
1063.49	917.44	9	s	5	520.25	666.31
1177.54	1031.48	10	N	4	433.22	579.27
1234.56	1088.50	11	G	3	319.17	465.23
1321.59	1175.53	12	S	2	262.15	408.21
1495.70	1349.64	13	R	1	175.12	

*Highlighted fragments were found in the MS^2 scan.

Supplementary Figure 7. HCD MS2 spectrum of the tryptic SPY peptide SPVVENGFs$^{O\text{-}Fuc}$NGSR (precursor m/z=748.352). SPY was transiently expressed in *N. benthamiana,* and *O*-fucosylated proteins were enriched by AAL pulldown. The HCD MS2 spectrum produces b and y ions sufficient enough to identify the peptide and even though there is loss of the *O*-linked fucose, there are y fragment ions that retain the fucose (denoted with an *) indicating the modified residue is S9. Observed ions are highlighted in the fragment mass table.

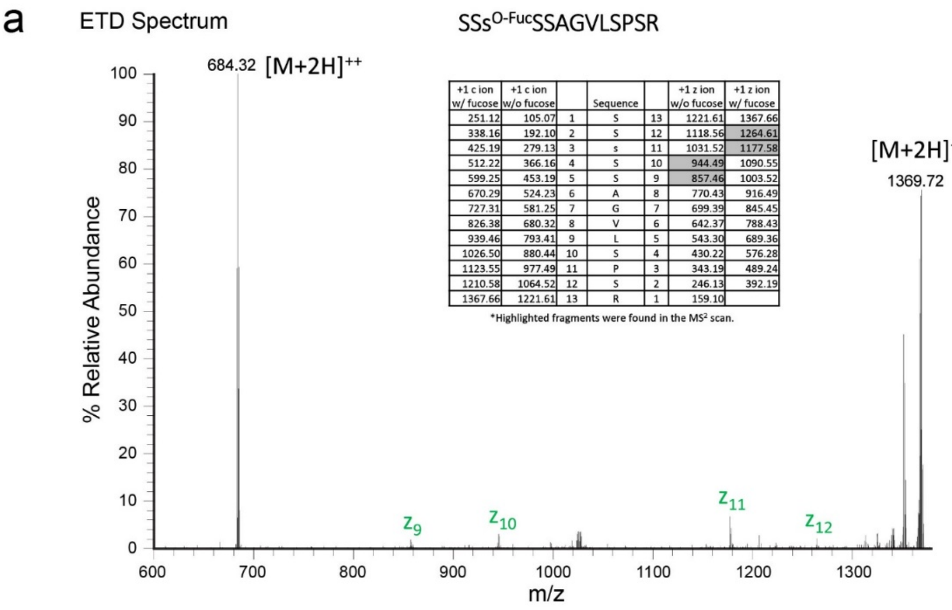

+1 c ion w/ fucose	+1 c ion w/o fucose		Sequence		+1 z ion w/o fucose	+1 z ion w/ fucose
251.12	105.07	1	S	13	1221.61	1367.66
338.16	192.10	2	S	12	1118.56	1264.61
425.19	279.13	3	s	11	1031.52	1177.58
512.22	366.16	4	S	10	944.49	1090.55
599.25	453.19	5	S	9	857.46	1003.52
670.29	524.23	6	A	8	770.43	916.49
727.31	581.25	7	G	7	699.39	845.45
826.38	680.32	8	V	6	642.37	788.43
939.46	793.41	9	L	5	543.30	689.36
1026.50	880.44	10	S	4	430.22	576.28
1123.55	977.49	11	P	3	343.19	489.24
1210.58	1064.52	12	S	2	246.13	392.19
1367.66	1221.61	13	R	1	159.10	

*Highlighted fragments were found in the MS^2 scan.

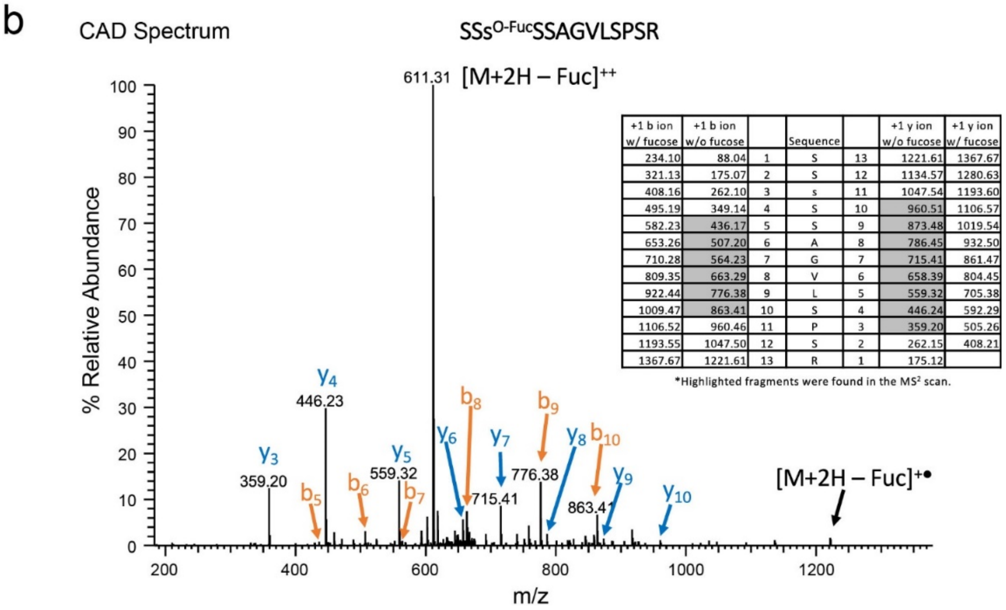

+1 b ion w/ fucose	+1 b ion w/o fucose		Sequence		+1 y ion w/o fucose	+1 y ion w/ fucose
234.10	88.04	1	S	13	1221.61	1367.67
321.13	175.07	2	S	12	1134.57	1280.63
408.16	262.10	3	s	11	1047.54	1193.60
495.19	349.14	4	S	10	960.51	1106.57
582.23	436.17	5	S	9	873.48	1019.54
653.26	507.20	6	A	8	786.45	932.50
710.28	564.23	7	G	7	715.41	861.47
809.35	663.29	8	V	6	658.39	804.45
922.44	776.38	9	L	5	559.32	705.38
1009.47	863.41	10	S	4	446.24	592.29
1106.52	960.46	11	P	3	359.20	505.26
1193.55	1047.50	12	S	2	262.15	408.21
1367.67	1221.61	13	R	1	175.12	

*Highlighted fragments were found in the MS^2 scan.

Supplementary Figure 8. ETD and CAD MS2 spectra of the tryptic SPY peptide SSs$^{O\text{-}Fuc}$SSAGVLSPSR (precursor m/z=684.334). SPY was transiently expressed in *N. benthamiana*, and *O*-fucosylated proteins were enriched by AAL pulldown. **a**, ETD MS2 spectrum indicates that Z9 and Z10 are not modified, while Z11 and Z12 are, indicating the modified residue is S3. **b**, The CAD MS2 spectrum produces b and y ions sufficient to identify the peptide and is dominated by the collisional dissociation loss of an O-linked fucose. Observed ions are highlighted in the respective fragment mass tables.

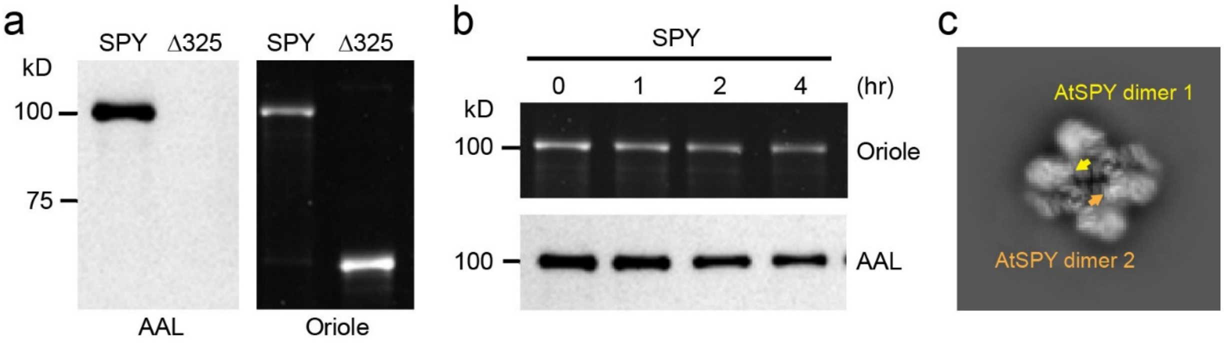

Supplementary Figure 9. *Trans*-auto-fucosylation of SPY. a, Full-length SPY purified from insect cells was *O*-fucosylated. Protein blot containing FL-SPY purified from insect cells and Δ325 purified from *E. coli* (a negative control) were probed with AAL-biotin followed by HRP-streptavidin. Oriole stained gel image shows similar loading. Representative images of 2 biological repeats are shown. **b,** Time course of *in vitro* POFUT assay using purified SPY from insect cells. No further auto-fucosylation was observed after 4 hr in the presence of GDP-fucose as detected by the AAL blot analysis. Representative images of 2 biological repeats are shown. **c**, A representative 2D class average of apo SPY particles showing a SPY tetramer formed by side-by-side stacking of two SPY dimers, revealing a possible mode of *trans*-auto-fucosylation. Arrows indicate locations of the N-terminal TPRs of individual SPY dimers and their proximity to the catalytic domain of the neighboring SPY dimer. Due to the small percentage of particles in the tetrameric state (~3% of the particles of the dimeric state), these particles were not selected for further 3D reconstruction at high resolution. Source data for **a-b** are provided as a Source Data file.

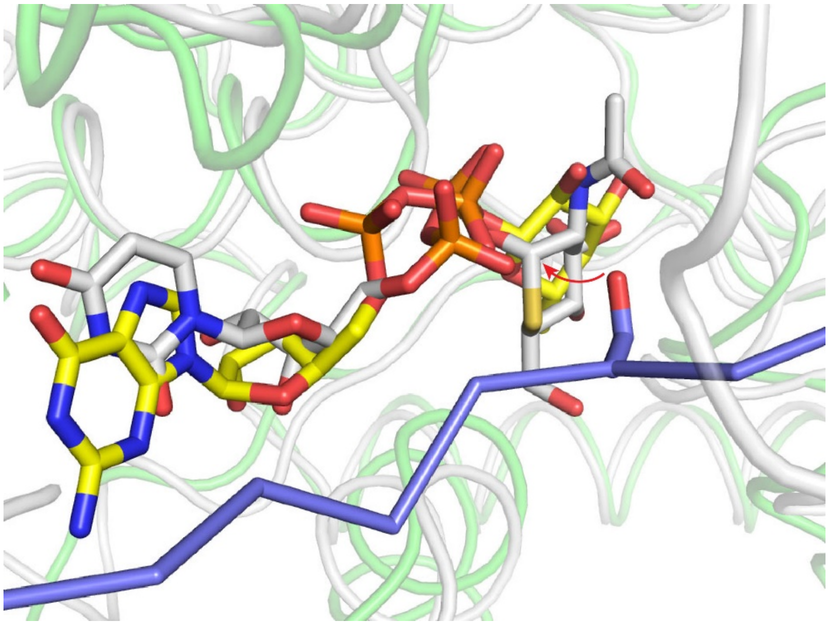

Supplementary Figure 10. Proposed catalytic mechanism of SPY. Superimposition of the catalytic domains of the GDP-fucose bound SPY and hOGT complexed with UDP-5S-GlcNAc and a substrate peptide (PDB: 4xif) shows that the sidechain of the substrate serine is well positioned for nucleophilic attack of the anomeric carbon of the GDP-fucose sugar donor. SPY and hOGT are shown in the cartoon loop model and are colored in green and grey, respectively. GDP-fucose and UDP-GlcNAc are shown in the stick model, and their carbon atoms are colored in yellow and grey, respectively. The substrate peptide of hOGT is shown in the Cα trace and colored in dark blue, with the sidechain of the receiving serine residue shown in the stick model.

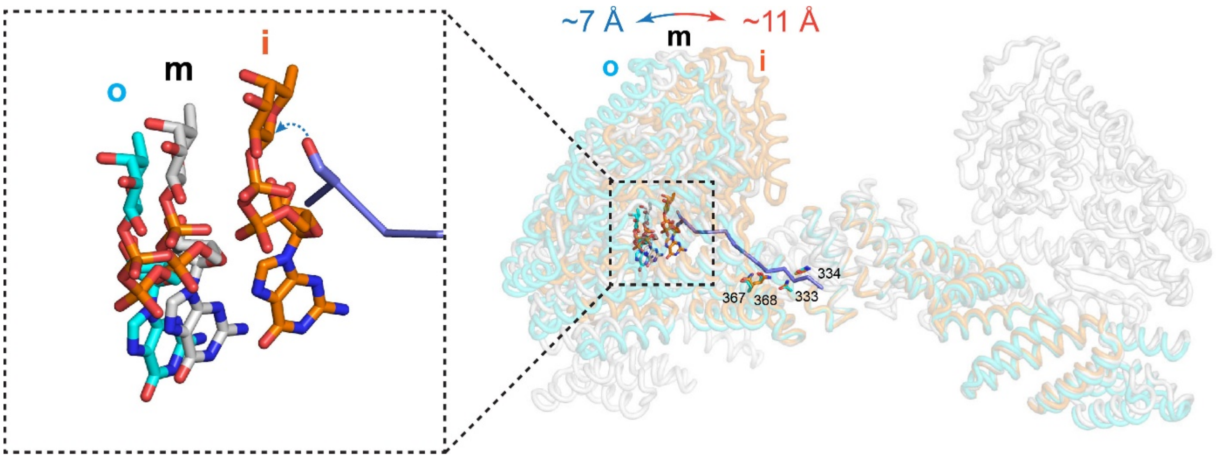

Supplementary Figure 11. Functional implication of the conformational dynamics of SPY. Apo SPY with the catalytic domain in different conformational states (o, outward; m, middle; i, inward) superimposed on central TPRs (TPR6-9; residue 218-359) are colored in cyan, grey, and orange, respectively. A substrate peptide (FSNGSRSSSSSAG, corresponding to F20-G32 of the auto-fucosylated N-terminal peptide of SPY, colored in purple) is modeled based on the backbone coordinate of the hOGT substrate peptide Tab1 (PDB: 5lvv) and shown in the Cα trace. The sidechain of the fucosylation acceptor serine residue is shown in the stick model. The putative Asn residues ("Asn ladder") for the substrate peptide backbone recognition are labeled. The GDP-fucose in the catalytic domain was generated by superimposing the catalytic domain of GDP-fucose bound SPY with the three conformations of the catalytic domain of apo SPY, illustrating the movement of GDP-fucose in these conformational states and their relative position to the acceptor serine residue.

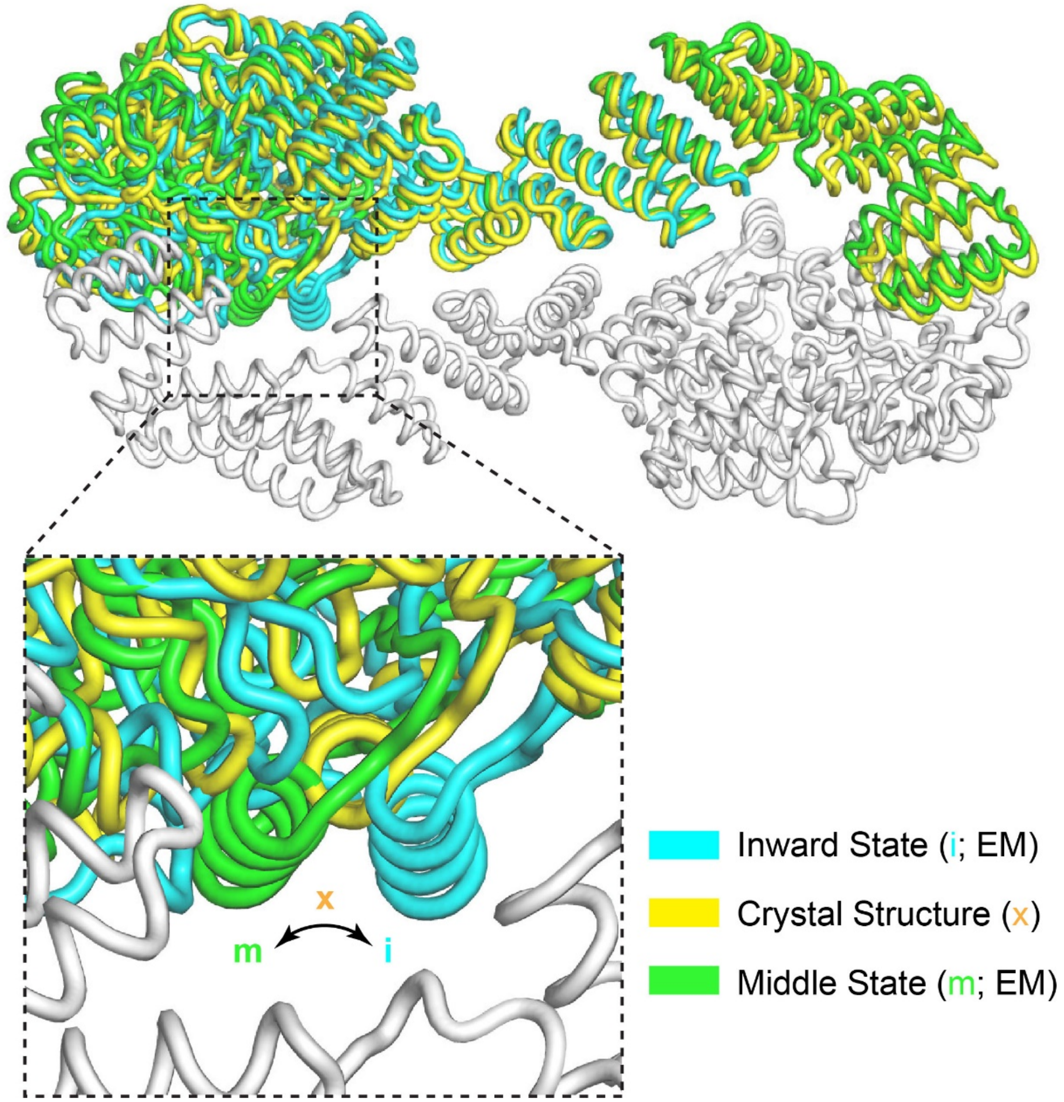

Supplementary Figure 12. Structural comparison of cryo-EM structure of Arabidopsis SPY and SPY/GDP-fucose complex with a recently reported crystal structure of SPY/GDP complex. The cryo-EM structure of the SPY/GDP-fucose complex with its catalytic domain in the inward conformation (PDB: 8DTI) is colored in cyan, the cryo-EM structure of apo SPY containing full TPRs with the catalytic domain in the middle state (PDB: 8DTF) is colored in green, and the crystal structure of the SPY/GDP complex (PDB: 7Y4I) is colored in yellow.

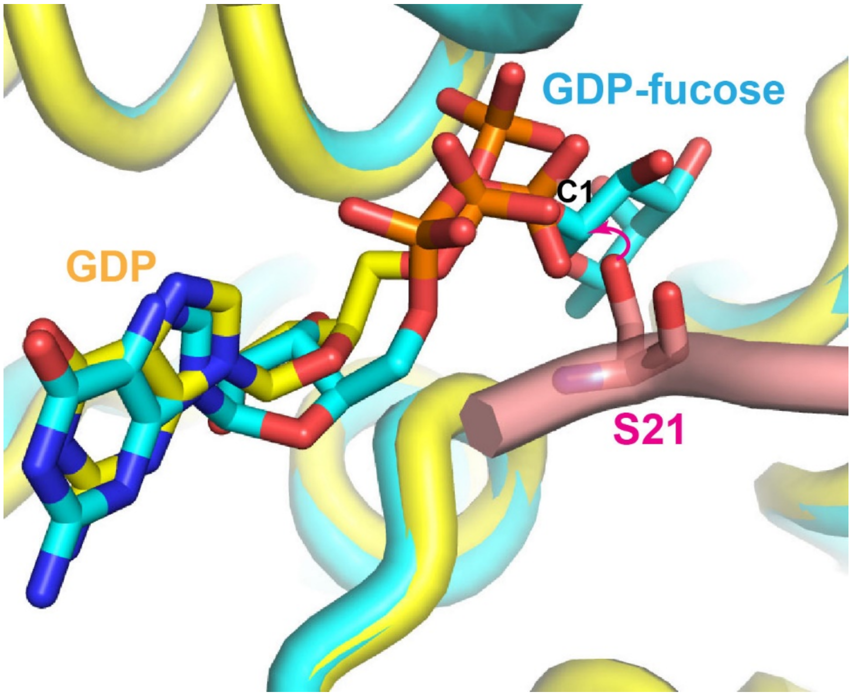

Supplementary Figure 13. Overlay of the catalytic domain of the cryo-EM structure of the SPY/GDP-fucose complex with the crystal structure of the self-inhibitory SPY/GDP complex reveals a substrate-based SN2 mechanism. The cryo-EM structure of the SPY/GDP-fucose complex (PDB: 8DTI) is colored in cyan, and the crystal structure of the self-inhibitory SPY/GDP complex (PDB: 7Y4I) is colored in yellow. The self-inhibitory N-terminal peptide of SPY is colored in light pink. GDP-fucose (cyan), GDP (yellow), and the substrate catalytic serine residue (S21, light pink) is shown in the stick model.

Supplementary Table 1. Cryo-EM data collection, refinement, and validation statistics of the full-length *Arabidopsis* SPY and SPY/GDP-Fucose complexes

	**Apo SPY Full TPR** (EMDB-27696) (PDB 8DTF)	**Apo SPY Alternative Conformation 1** (EMDB-27697) (PDB 8DTG)	**Apo SPY Alternative Conformation 2** (EMDB-27698) (PDB 8DTH)	**SPY/GDP-Fucose complex** (EMDB-27700)[#] (EMDB-27699)[†] (PDB 8DTI)
**Data collection and processing**				
Magnification	54,900	54,900	54,900	81,000
Voltage (kV)	200	200	200	300
Electron exposure (e–/Å^2)	55	55	55	52.4
Defocus range (μm)	[-2.0, -0.5]	[-2.0, -0.5]	[-2.0, -0.5]	[-2.0, -1.0]
Pixel size (Å)	0.880	0.880	0.880	1.080
Symmetry imposed	C2	C1	C1	C2
Initial particle images (no.)	~8 M	~8 M	~8 M	~4 M
Final particle images (no.)	91,837	80,304	269,690	182,947
Map resolution (Å)	3.7	3.9	3.6	3.8[#]/3.6[†]
FSC threshold	0.143	0.143	0.143	0.143
Map resolution range (Å)	3.2-6.4	3.2-6.4	3.0-6.4	3.5-5.5
**Refinement**				
Initial model used (PDB code)	N/A	N/A	N/A	N/A
Model resolution (Å)	3.6	3.7	3.4	3.4
FSC threshold	0.143	0.143	0.143	0.143
Model resolution range (Å)	3.2-6.4	3.2-6.4	3.0-6.4	3.5-5.5
Map sharpening *B* factor (Å^2)	-122	-121	-129	-204
Model composition				
Non-hydrogen atoms	12,414	11,451	11,153	9,455
Protein residues	1,606	1,539	1,437	1,258
Ligands	0	0	0	2
B factors (Å^2)				
Protein	77.74	97.34	84.55	72.89
Ligand				71.81
R.m.s. deviations				
Bond lengths (Å)	0.003	0.004	0.003	0.003
Bond angles (°)	0.726	0.637	0.612	0.513
Validation				
MolProbity score	1.78	1.81	1.55	1.40
Clashscore	8.95	7.78	3.81	4.70
Rotamer outliers (%)	0.00	0.00	0.00	0.00
Ramachandran plot				
Favoured (%)	95.63	94.40	94.49	97.13
Allowed (%)	4.37	5.60	5.51	2.87
Disallowed (%)	0.00	0.00	0.00	0.00

[#]Overall map
[†]Composite map

Supplementary Table 2. Key residues of the SPY-Like, intermediate, and OGT/SEC-Like glycosyltransferases of the GT41 family

		AtSPY K689	AtSPY A731	AtSPY Y744
**SPY-Like**	RHIZ_Bigelowiella_natans_CCMP2755_phycocosm|jgi|Bigna1|18628|gw1.59.4.1	K	L	Y
	ALVE_Toxoplasma_gondii_ME49_phycocosm|jgi|Toxgon1|3897|XM_002365910.1	K	V	Y
	green_Arabidopsis_thaliana_TAIR10|AT3G11540.1	K	A	Y
	green_Chlamydomonas_reinhardtii_v5_6_phycocosm|jgi|Chlre5_6|4732|Cre12.g552851.t1.1	K	Q	Y
	green_Chloropicon_primus_CCMP1205_phycocosm|jgi|Chlpri1|7884|rna-gnl|IITBIO|A3770_17p78840_mRNA_A3770_17p78840	K	V	Y
	green_Coccomyxa_sp_C169_phycocosm|jgi|Coc_C169_1|35165|fgenesh1_pm.2_#_175	K	T	Y
	green_Klebsormidium_nitens|kfl00136_0200_v1.1	K	S	Y
	green_Micromonas_pusilla_CCMP1545_phytozome|46435	K	M	Y
	green_Ostreococcus_lucimarinus_phytozome|863	K	M	Y
	green_Volvox_carteri_v2_1_phycocosm|jgi|Volca2_1|14539|Vocar.0007s0325.1	K	T	Y
	red_Galdieria_sulphuraria_074W_phycocosm|jgi|Galsul1|4573|XM_005704541.1	K	T	Y
	red_Gracilariopsis_chorda_Rutgers|PXF45348.1	K	A	Y
	red_Porphyridium_purpureum_v2_1_Rutgers|POR8187_scf295_1	K	M	Y
	red_Porphyridium_purpureum_v2_1_Rutgers|POR7139_scf209_3	K	M	Y
	green_AagrOXF_evm.model.utg000013l.168.1	K	A	Y
	green_Atrichopoda_AmTr_v1.0_scaffold00040.99	K	A	Y
	green_Azolla_filiculoides_Azfi_s0167.g054517	K	A	Y
	green_Azolla_filiculoides_Azfi_s0617.g079537	K	A	Y
	green_Cbr_1_0_genomic_pep_rna-CBR_g30524	K	S	Y
	green_Mguttatus_Migut.J01002.1	K	A	Y
	green_Mpolymorpha_Mapoly0020s0107.1	K	A	Y
	green_Mtruncatula_Medtr1g059610.1	K	A	Y
	green_Osativa_LOC_Os08g44510.1	K	A	Y
	green_Ppatens_Pp3c2_30810V3.1	K	A	Y
	green_Ppatens_Pp3c1_2450V3.1	K	A	Y
	green_Ppatens_Pp3c1_2460V3.1	K	A	Y
	green_S_lep_V4_2_pep_maker-Contig_28-snap-gene-3.83-mRNA-1	K	S	Y
	green_Salvinia_cucullata_Sacu_v1.1_s0016.g006858	K	A	Y
	green_Sfallax_Sphfalx17G028800.1	K	A	Y
	green_Slycopersicum_Solyc09g010180.2.1	K	A	Y
	green_Smoellendorffii_159012	K	A	Y
	green_Vvinifera_GSVIVT01033891001	K	A	Y
	green_Zmays_Zm00001d031938_T001	K	A	Y
	Micromonas_commoda|ACO61142.1	K	V	Y
	Dictyostelium_discoideum_AX4|XP_638298.1	K	Y	Y
	Rhodospirillum_rubrum_ATCC_11170|YP_427939.1	K	V	Y
	Bradyrhizobium_diazoefficiens_USDA_110|NP_771090.1	K	A	Q
	Burkholderia_lata|YP_367586.1	K	A	Y
	Burkholderia_sp._H160|ZP_03265964.1	A	S	Y
	Methylococcus_capsulatus_str._Bath|YP_113965.1	Q	T	F
**Intermediate**	Desulfococcus_oleovorans_Hxd3|YP_001528971.1	S	R	Y
	Azorhizobium_caulinodans_ORS_571|YP_001526382.1	Y	R	Y
	STRbrown_Cladosiphon_okamuranus|g6607.t1	L	R	Y
	STRoomy_Phytophthora_infestans_T30-4_phycocosm|jgi|Phyinf1|874|rna18349	L	R	Y
	STRchryso_Ochromonadaceae_sp_CCMP2298_phycocosm|jgi|Ochro2298_1|192132|CE192131_6500	M	R	Y
	STRbrown_Cladosiphon_okamuranus|g12363.t1	L	R	Y
	STRoomy_Saprolegnia_parasitica_CBS223_65_phycocosm|jgi|Sappar1|24620|rna5580	L	R	Y
	STRbrown_Cladosiphon_okamuranus|g10582.t1	L	R	Y
	STRpelago_Aureococcus_anophagefferens_phycocosm|jgi|Auran1|64890	L	R	Y
	STRpelago_Pelagophyceae_sp_CCMP2097_phycocosm|jgi|Pelago2097_1|505891|fgenesh1_kg.3_#_831_#_Locus25271v1rpkm0.91	L	R	Y
	STRbrown_Undaria_pinnatifida|HiC_scaffold_30.g13371.m1	L	R	Y
**OGT/SEC-Like**	red_Condrus_cripus_Stackhouse_phycocosm|jgi|Chocri1|3740|XM_005715719.1	L	R	C
	red_Galdieria_sulphuraria_074W_phycocosm|jgi|Galsul1|384|XM_005707912.1	L	R	C
	red_Gracilariopsis_chorda_Rutgers|PXF46574.1	L	R	C
	red_Porphyra_umbilicalis_4086291_phycocosm|jgi|Porumb1|5158|rna8075	L	R	C
	red_Porphyra_umbilicalis_4086291_phycocosm|jgi|Porumb1|5159|rna8076	L	R	C
	STRoomy_Saprolegnia_parasitica_CBS223_65_phycocosm|jgi|Sappar1|31175|rna9122	L	R	Y
	STRpelago_Aureococcus_anophagefferens_phycocosm|jgi|Auran1|316	H	H	C
	STRbrown_Cladosiphon_okamuranus|g14006.t1	L	R	C
	STRbrown_Ectocarpus_siliculosus_phycocosm|jgi|Ectsil1|31348|rna2136	L	R	C
	STRbrown_Nemacystus_decipiens|g2365.t1	L	R	C
	STRbrown_Undaria_pinnatifida|HiC_scaffold_22.g09886.m1	L	R	C
	STRoomy_Phytophthora_infestans_T30-4_phycocosm|jgi|Phyinf1|15199|rna2986	L	R	C
	STRpelago_Aureococcus_anophagefferens_phycocosm|jgi|Auran1|359	L	R	C
	green_Arabidopsis_thaliana_TAIR10|AT3G04240.1	L	R	C
	green_Klebsormidium_nitens|kfl00945_0020_v1.1	L	R	C
	green_Prasinoderma_coloniale|PRCOL_00000371-RA	L	R	Y
	green_Mesotaenium_endlicherianum|ME000444S07396	A	R	C
	green_AagrOXF_evm.model.utg000103l.193.1	L	R	C
	green_Mguttatus_Migut.G01152.1	L	R	C
	green_Mguttatus_Migut.G00821.1	L	R	C
	STRpelago_Aureococcus_anophagefferens_phycocosm|jgi|Auran1|54803	I	R	C
	green_Mtruncatula_Medtr1g079510.1	L	R	C
	green_Atrichopoda_AmTr_v1.0_scaffold00013.214	L	R	C
	green_Azolla_filiculoides_Azfi_s0702.g082634	L	R	C
	green_Cbr_1_0_genomic_pep_rna-CBR_g46307	L	R	C
	green_Mpolymorpha_Mapoly0034s0037.1	L	R	C
	green_Osativa_LOC_Os01g68680.1	L	R	C
	green_Ppatens_Pp3c5_10370V3.1	L	R	C
	green_Ppatens_Pp3c6_13940V3.1	L	R	C

	green_S_lep_V4_2_pep_maker-Contig_282-snap-gene-0.54-mRNA-1	L	R	C
	green_Salvinia_cucullata_Sacu_v1.1_s0070.g016668	L	R	C
	green_Sfallax_Sphfalx05G071200.1	L	R	C
	green_Sfallax_Sphfalx02G070200.1	L	R	C
	green_Sfallax_Sphfalx01G081800.1	L	R	C
	green_Slycopersicum_Solyc09g097830.2.1	L	R	C
	green_Smoellendorffii_143846	L	R	C
	green_Vvinifera_GSVIVT01026359001	L	R	C
	green_Zmays_Zm00001d016506_T003	L	R	C
	Anopheles_gambiae_str._PEST|XP_316319.4	L	R	C
	Aspergillus_nidulans_FGSC_A4|XP_657869.1	L	R	C
	Caenorhabditis_elegans|NP_001040860.1	L	R	C
	Canis_familiaris|XP_858769.1	L	R	C
	Ciona_intestinalis|XP_002131769.2	L	R	C
	Danio_rerio|NP_001017359.1	L	R	C
	Drosophila_melanogaster|NP_523620.1	L	R	C
	Homo_sapiens|NP_858058.1	L	R	C
	Neurospora_crassa|CAC28786.1	L	R	C
	Rattus_norvegicus|EDL95885.1	L	R	C
	Strongylocentrotus_purpuratus|XP_784504.3	L	R	C
	Ustilago_maydis_521|XP_757772.1	L	R	C
	Xenopus_laevis|NP_001087833.1	L	R	C
	Acyrthosiphon_pisum|XP_001952129.2	L	R	C

Supplementary Table 3. Primers and their uses

PRIMER NAME	SEQUENCE (5' -> 3')	USE	NOTES
attB1-site	GGGGACAAGTTTGTACAAAAAAGCAGGCT	PCR inserts in pDONR-221 to recombine into pDONR207	Construction of pDONR207-SPY point mutations
attB2-site	GGGGACCACTTTGTACAAGAAAGCTGGGT		
AtSPY-△1-39aa-F	GGGGACAAGTTTGTACAAAAAAGCAGGCTACATG GTCACTCAGGGGAACGATACAC	For cloning SPY deletion to pDONR207	Construction of pDONR207-SPY △1-39aa
AtSPY-△1-216aa-F	GGGGACAAGTTTGTACAAAAAAGCAGGCTACATG CCTATGTATGCTGAAGCATATTG		Construction of pDONR207-SPY△1-216aa
SPY L320A F324A-F	ATTGTCTTCTATGAGGCGGCTTTCCACGCGAATCCACATTGTGCT	For cloning SPYm2 to pDONR207	Construction of pDONR207-SPYm2
SPY L320A F324A-R	AGCACAATGTGGATTCGCGTGGAAAGCCGCCTCATAGAAGACAAT		
SPY W292A-F	GCTCTCTATTATAACGCGCACTATGCAGATGCT	For cloning SPYm3 to pDONR207	Construction of pDONR207-SPYm3
SPY W292A-R	AGCATCTGCATAGTGCGCGTTATAATAGAGAGC		
SPY N662A-F	TTCAACgccCTCGCAAAGATAACTCCTAAGGTGCTG	For cloning SPY N662A to pDONR221	Construction of pDONR221-SPY N662A
SPY N662A-R	TGCGAGggcGTTGAAACTACCAAATGTGACAAAGCC		
SPY K665A-F	CTCGCAgccATAACTCCTAAGGTGCTGCAAGTGTG	For cloning SPY K665A to pDONR221	Construction of pDONR221-SPY K665A
SPY K665A-R	AGTTATggcTGCGAGGTTGTTGAAACTACCAAATGTG		
SPY K689A-F	GTGGTAgccTGCAAACCTTTCTGCTGCGAT	For cloning SPY K689A to pDONR221	Construction of pDONR221-SPY K689A
SPY K689A-R	GTTTGCAggcTACCACTAGACGAGAATTGGGA		
SPY H728A-F	CACGACgccATGCAAGCCTATTCCTTGATGGATATTAGTTTG	For cloning SPY H728A to pDONR221	Construction of pDONR221-SPY H728A
SPY H728A-R	TTGCATggcGTCGTGATTGAAAAGAATCAAAGGCAAGAG		
SPY Y744A-F	TTCCCTgccGCTGGAACTACCACTACCTGTGAG	For cloning SPY Y744A to pDONR221	Construction of pDONR221-SPY Y744A
SPY Y744A-R	TCCAGCggcAGGGAATGTGTCCAAACTAATATCCATC		
SPY T747A-F	GCTGGAgccACCACTACCTGTGAGTCTCTCTACATG	For cloning SPY T747A to pDONR221	Construction of pDONR221-SPY T747A
SPY T747A-R	AGTGGTggcTCCAGCATAAGGGAATGTGTCCAAAC		
SPY S496A-F	GATTTCTTCACTCAT GCA GTATCTTATTTCATT	For cloning SPY S496A to pDONR221	Construction of pDONR221-SPY S496A
SPY S496A-R	AATGAAATAAGATACTGCATGAGTGAAGAAATC		
SPY V497R-F	TTCTTCACTCATTCAAGATCTTATTTCATTGAA	For cloning SPY V497R to pDONR221	Construction of pDONR221-SPY V497R
SPY V497R-R	TTCAATGAAATAAGATCTTGAATGAGTGAAGAA]		
SPY I592R-F	GTTCAGGTTACTTGG AGA GGCTATCCAAATACT	For cloning SPY I592R to pDONR221	Construction of pDONR221-SPY I592R
SPY I592R-R	AGTATTTGGATAGCCTCTCCAAGTAACCTGAAC		
SPY T748A-F	CCTTATGCTGGAACT GCCACTACCTGTGAGTCT	For cloning SPY T748A to pDONR221	Construction of pDONR221-SPY T748A
SPY T748A-R	AGACTCACAGGTAGTGGCAGTTCCAGCATAAGG		
SPY E752A-F	ACTACCACTACCTGT GCG TCTCTCTACATGGGA	For cloning SPY E752A to pDONR221	Construction of pDONR221-SPY E752A
SPY E752A-R	TCCCATGTAGAGAGACGCACAGGTAGTGGTAGT		
SPY_FL-FP	GTCCGAAGCGCGCGGAATTCATGGTGGGACTGGAAGATGATACTG	Cloning into FastBac1	
SPY_FL_RP1	GGTGTGAACCACCTTGGAAGTACAGGTTCTCGCTACCACCGCTAGT GGAGTCCATTCTCTTTG		
SPY_FL_RP2	TAGTACTTCTCGACAAGCTTTTATTTTTCGAACTGCGGGTGAGACCA ACCACCGTGATGGTGATGGTGATGGTGATGGTGGTGTGAACCACCTTGGAAGT		

Supplementary Table 4. List of constructs

Use	Construct	Plasmid name	Insert or PCR product	Primers	Template or Origin	Plasmid backbone	Cloning method
Entry clones	pDONR221-SPY N662A	pDONR221-SPY N662A	SPY N662A cDNA	N662A-F/ N662A-R	pDONR221-SPY	pDONR221	Infusion
	pDONR221-SPY K665A	pDONR221-SPY K665A	SPY K665A cDNA	K665A-F/ K665A-R	pDONR221-SPY		Infusion
	pDONR221-SPY K689A	pDONR221-SPY K689A	SPY K689A cDNA	K689A-F/ K689A-R	pDONR221-SPY		Infusion
	pDONR221-SPY H728A	pDONR221-SPY H728A	SPY H728A cDNA	H728A-F/ H728A-R	pDONR221-SPY		Infusion
	pDONR221-SPY Y744A	pDONR221-SPY Y744A	SPY Y744A cDNA	Y744A-F/ Y744A-R	pDONR221-SPY		Infusion
	pDONR221-SPY T747A	pDONR221-SPY T747A	SPY T747A cDNA	T747A-F/ T747A-R	pDONR221-SPY		Infusion
	pDONR207-SPY N662A	pDONR207-SPY N662A	SPY N662A cDNA	attB1-site/attB2-site	pDONR221-SPY N662A	pDONR207	Gateway BP
	pDONR207-SPY K665A	pDONR207-SPY K665A	SPY K665A cDNA	attB1-site/attB2-site	pDONR221-SPY K665A		
	pDONR207-SPY K689A	pDONR207-SPY K689A	SPY K689A cDNA	attB1-site/attB2-site	pDONR221-SPY K689A		
	pDONR207-SPY H728A	pDONR207-SPY H728A	SPY H728A cDNA	attB1-site/attB2-site	pDONR221-SPY H728A		
	pDONR207-SPY Y744A	pDONR207-SPY Y744A	SPY Y744A cDNA	attB1-site/attB2-site	pDONR221-SPY Y744A		
	pDONR207-SPY T747A	pDONR207-SPY T747A	SPY T747A cDNA	attB1-site/attB2-site	pDONR221-SPY T747A		
	pDONR207-SPY S496A	pDONR207-SPY S496A	SPY S496A cDNA	attB1-site/SPY S496A-R<br>SPY S496A-F/attB2-site**	pDONR207-SPY		
	pDONR207-SPY V497R	pDONR207-SPY V497R	SPY V497R cDNA	attB1-site/SPY V497R-R<br>SPY V497R-F/attB2-site**	pDONR207-SPY		
	pDONR207-SPY I592R	pDONR207-SPY I592R	SPY I592R cDNA	attB1-site/SPY I592R-R<br>SPY I592R-F/attB2-site**	pDONR207-SPY		
	pDONR207-SPY T748A	pDONR207-SPY T748A	SPY T748A cDNA	attB1-site/SPY T748A-R<br>SPY T748A-F/attB2-site**	pDONR207-SPY		
	pDONR207-SPY E752A	pDONR207-SPY E752A	SPY E752A cDNA	attB1-site/SPY E752A-R<br>SPY E752A-F/attB2-site**	pDONR207-SPY		
	pDONR-SPY	pDONR207-SPY	SPY cDNA	attB1-site/attB2-site	pDONR221-SPY		
	pDONR-SPYm2	pDONR207-SPYm2	SPYm2 cDNA	attB1-site/SPY L320A F324A-R<br>SPY L320A F324A-F/attB2-site**	pDONR207-SPY		
	pDONR-SPYm3	pDONR207-SPYm3	SPYm3 cDNA	attB1-site/SPY W292-R<br>SPY W292-F/attB2-site**	pDONR207-SPYm2		
	pDONR-SPY △1-39aa	pDONR-SPY △1-39aa	SPY △1-39aa cDNA	SPY △1-39aa-F/SPY attB2-site	pDONR207-SPY		
	pDONR-SPY ∆1-216aa	pDONR207-SPY ∆1-216aa	SPY ∆1-216aa cDNA	SPY ∆1-216aa-F/SPY attB2-site	pDONR207-SPY		
Transient expression	P_{35S}:Myc-SPY N662A	pEG203-SPY N662A	SPY N662A cDNA	-	pDONR207-SPY N662A	pEarleyGate203[##]	Gateway LR
	P_{35S}:Myc-SPY K665A	pEG203-SPY K665A	SPY K665A cDNA	-	pDONR207-SPY K665A		
	P_{35S}:Myc-SPY K689A	pEG203-SPY K689A	SPY K689A cDNA	-	pDONR207-SPY K689A		
	P_{35S}:Myc-SPY H728A	pEG203-SPY H728A	SPY H728A cDNA	-	pDONR207-SPY H728A		
	P_{35S}:Myc-SPY Y744A	pEG203-SPY Y744A	SPY Y744A cDNA	-	pDONR207-SPY Y744A		
	P_{35S}:Myc-SPY T747A	pEG203-SPY T747A	SPY T747A cDNA	-	pDONR207-SPY T747A		
	P_{35S}:Myc-SPY S496A	pEG203-SPY S496A	SPY S496A cDNA	-	pDONR207-SPY S496A		
	P_{35S}:Myc-SPY V497R	pEG203-SPY V497R	SPY V497R cDNA	-	pDONR207-SPY V497R		
	P_{35S}:Myc-SPY I592R	pEG203-SPY I592R	SPY I592R cDNA	-	pDONR207-SPY I592R		
	P_{35S}:Myc-SPY T748A	pEG203-SPY T748A	SPY T748A cDNA	-	pDONR207-SPY T748A		

	P_{35S}:Myc-SPY E752A	pEG203-SPY E752A	SPY E752A cDNA	-	pDONR207-SPY E752A		
	P_{35S}:Myc-SPYm2	pEG203-SPYm2	SPYm2 cDNA	-	pDONR207-SPYm2		
	P_{35S}:Myc-SPYm3	pEG203-SPYm3	SPYm3 cDNA	-	pDONR207-SPYm3		
	P_{35S}:Myc-SPY △1-39aa	pEG203-SPY △1-39aa	SPY △1-39aa cDNA	-	pDONR207-SPY △1-39aa		
	P_{35S}:Myc-SPY ∆1-216aa	pEG203-SPY ∆1-216aa	SPY ∆1-216aa cDNA	-	pDONR207-SPY ∆1-216aa		
	P_{35S}:Myc-SPY	pEG203-SPY[#]	SPY cDNA	-	pDONR221-SPY		
	P_{35S}:Myc-GUS	pEG203-GUS[#]	GUS cDNA		pDONR221-GUS		
	P_{35S}:FLAG-RGA*	pEG100-3xFR[#]	RGA cDNA		pDONR-3xFR	pEarleyGate100[##]	

* Construct P_{35S}:FLAG-RGA contains a 6xHis-3xFLAG tag for tandem affinity purification.

**PCR products of SPY m2/m3 cDNA were obtained by overlapping PCR.

[#] Zentella, R. *et al.* (2016)[1]

[##] Earley, K. W. *et al.* (2006)[2]

[###] Zentella, R. *et al.* (2017)[3]

SUPPLEMENTARY REFERENCES

1 Zentella, R. *et al.* *O*-GlcNAcylation of master growth repressor DELLA by SECRET AGENT modulates multiple signaling pathways in Arabidopsis. *Genes Dev* **30**, 164-176 (2016).

2 Earley, K. W. *et al.* Gateway-compatible vectors for plant functional genomics and proteomics. *Plant J* **45**, 616-629 (2006).

3 Zentella, R. *et al.* The Arabidopsis *O*-fucosyltransferase SPINDLY activates nuclear growth repressor DELLA. *Nat Chem Biol* **13**, 479-485 (2017).
