# Supplementary material for: Structure and dynamics of the Arabidopsis O-fucosyltransferase SPINDLY
Source: Nat Commun. 2023 Mar 20;14:1538. doi: 10.1038/s41467-023-37279-1 (PMC10027727; doi:10.1038/s41467-023-37279-1)
Supplement: Supplementary file 2 — Reporting Summary [file 41467_2023_37279_MOESM2_ESM.pdf]

nature portfolio

Corresponding author(s): Pei Zhou, Tai-Ping Sun, Alberto Bartesaghi

Last updated by author(s): 2/21/2023

# Reporting Summary

Nature Portfolio wishes to improve the reproducibility of the work that we publish. This form provides structure for consistency and transparency in reporting. For further information on Nature Portfolio policies, see our Editorial Policies and the Editorial Policy Checklist.

## Statistics

For all statistical analyses, confirm that the following items are present in the figure legend, table legend, main text, or Methods section.

| n/a | Confirmed | |
|---|---|---|
| ☐ | ☒ | The exact sample size (*n*) for each experimental group/condition, given as a discrete number and unit of measurement |
| ☐ | ☒ | A statement on whether measurements were taken from distinct samples or whether the same sample was measured repeatedly |
| ☒ | ☐ | The statistical test(s) used AND whether they are one- or two-sided<br>*Only common tests should be described solely by name; describe more complex techniques in the Methods section.* |
| ☒ | ☐ | A description of all covariates tested |
| ☒ | ☐ | A description of any assumptions or corrections, such as tests of normality and adjustment for multiple comparisons |
| ☒ | ☐ | A full description of the statistical parameters including central tendency (e.g. means) or other basic estimates (e.g. regression coefficient) AND variation (e.g. standard deviation) or associated estimates of uncertainty (e.g. confidence intervals) |
| ☒ | ☐ | For null hypothesis testing, the test statistic (e.g. *F*, *t*, *r*) with confidence intervals, effect sizes, degrees of freedom and *P* value noted<br>*Give P values as exact values whenever suitable.* |
| ☒ | ☐ | For Bayesian analysis, information on the choice of priors and Markov chain Monte Carlo settings |
| ☒ | ☐ | For hierarchical and complex designs, identification of the appropriate level for tests and full reporting of outcomes |
| ☒ | ☐ | Estimates of effect sizes (e.g. Cohen's *d*, Pearson's *r*), indicating how they were calculated |

*Our web collection on statistics for biologists contains articles on many of the points above.*

## Software and code

Policy information about availability of computer code

| | |
|---|---|
| Data collection | Cryo-EM data were collected using the SerialEM software (version 3.8.7) and Latitude S (Version 3.51.3719.0). |
| Data analysis | Cryo-EM data analysis was conducted using CTFFIND4 (version 4.1.13) and cryoSPARC (version 3.3.1). Model building and refinement for cryoEM and crystal structures were carried out using COOT (version 0.8.9.2) and PHENIX (version 1.19.2-4158), respectively. Phylogenetic analysis was carried out using Orthofinder v2.5.4, MAFFT v7, trimAl v1.4 and IQ-TREE v2.0.3, and the resulting tree was plotted by iTOL V6. |

For manuscripts utilizing custom algorithms or software that are central to the research but not yet described in published literature, software must be made available to editors and reviewers. We strongly encourage code deposition in a community repository (e.g. GitHub). See the Nature Portfolio guidelines for submitting code & software for further information.

## Data

Policy information about availability of data

All manuscripts must include a data availability statement. This statement should provide the following information, where applicable:

- Accession codes, unique identifiers, or web links for publicly available datasets
- A description of any restrictions on data availability
- For clinical datasets or third party data, please ensure that the statement adheres to our policy

The cryo-EM structures of apo SPY in three conformations and the SPY/GDP-fucose complex have been deposited to the Protein Data Bank (www.pdb.org) with access codes of 8DTF [https://www.rcsb.org/structure/8DTF], 8DTG [https://www.rcsb.org/structure/8DTG], 8DTH [https://www.rcsb.org/structure/8DTH], and 8DTI [https://www.rcsb.org/structure/8DTI]. The three corresponding maps of apo SPY and the overall and composite maps of the SPY/GDP-fucose complex have been deposited to EMDB under the access codes of
EMD-27696 [https://www.emdataresource.org/EMD-27696],
EMD-27697 [https://www.emdataresource.org/EMD-27697],
EMD-27698 [https://www.emdataresource.org/EMD-27698],
EMD-27700 [https://www.emdataresource.org/EMD-27700],
and EMD-27699 [https://www.emdataresource.org/EMD-27699], respectively.
The mass spectrometry proteomics data have been deposited to the ProteomeXchange Consortium via the PRIDE 57 partner repository with the dataset identifier PXD040480 [Project DOI: 10.6019/PXD040480].
Source data are provided with this paper.

## Human research participants

Policy information about studies involving human research participants and Sex and Gender in Research.

| | |
|---|---|
| Reporting on sex and gender | not applicable |
| Population characteristics | not applicable |
| Recruitment | not applicable |
| Ethics oversight | not applicable |

Note that full information on the approval of the study protocol must also be provided in the manuscript.

# Field-specific reporting

Please select the one below that is the best fit for your research. If you are not sure, read the appropriate sections before making your selection.

☒ Life sciences ☐ Behavioural & social sciences ☐ Ecological, evolutionary & environmental sciences

For a reference copy of the document with all sections, see nature.com/documents/nr-reporting-summary-flat.pdf

# Life sciences study design

All studies must disclose on these points even when the disclosure is negative.

| | |
|---|---|
| Sample size | For agroinfiltration assays, 8 leaves (from 4 plants) were used for each sample in a biological repeat. |
| Data exclusions | No data were excluded from analysis. |
| Replication | All protein gel blot analyses were repeated two to three times (biological repeats). |
| Randomization | Location of plants for agroinfiltration was randomized. |
| Blinding | Blinding was not applied because specific combinations of constructs need to be used for different samples in our assays. |

# Reporting for specific materials, systems and methods

We require information from authors about some types of materials, experimental systems and methods used in many studies. Here, indicate whether each material, system or method listed is relevant to your study. If you are not sure if a list item applies to your research, read the appropriate section before selecting a response.

## Materials & experimental systems

| n/a | Involved in the study |
|---|---|
| ☐ | ☒ Antibodies |
| ☐ | ☒ Eukaryotic cell lines |
| ☒ | ☐ Palaeontology and archaeology |
| ☒ | ☐ Animals and other organisms |
| ☒ | ☐ Clinical data |
| ☒ | ☐ Dual use research of concern |

## Methods

| n/a | Involved in the study |
|---|---|
| ☒ | ☐ ChIP-seq |
| ☒ | ☐ Flow cytometry |
| ☒ | ☐ MRI-based neuroimaging |

# Antibodies

**Antibodies used**

HRP-conjugated anti-FLAG mouse monoclonal antibody (M2 clone, Sigma-Aldrich A8592) was used to detect FLAG-RGA.
HRP-conjugated anti-cMyc rabbit polyclonal antibody (Sigma-Aldrich, A5598) was used to detect Myc-SPY.
Anti-FLAG agarose (Sigma-Aldrich, A2220) was used for immunoprecipitation of FLAG-tagged proteins.
Anti-Myc agarose (Sigma-Aldrich, A7470) was used for immunoprecipitation of Myc-tagged proteins.
Fucose-specific lectin (not an antibody): Biotinylated-Aleuria aurantia lectin (ML-biotin, Vector Labs, B-1395).
Streptavidin-HRP (Jackson Immunoresearch Labs, Cat. 016-030-084)

**Validation**

Sigma-Aldrich A8592 has minimum detection of 8 ng of FLAG-fusion protein N-BAP (sigmaaldrich.com).
Sigma-Aldrich A5598: A minimum working dilution of 1:5000 is determined by immunoblotting of an E. coli extract which expresses a recombinant c-Myc tagged fusion protein (sigmaaldrich.com).
Sigma-Aldrich A7470 binding capacity: 1 mL of settled beads has a binding capacity of ≥ 10 nmole of c-Myc-tagged fusion protein (sigmaaldrich.com).
Sigma-Aldrich A2220: ≥ 0.6mg N-BAP eluted per ml of packed resin (sigmaaldrich.com).
In all our experiments, we included negative and positive controls to ensure the specificity of each antibody or lectin

# Eukaryotic cell lines

Policy information about cell lines and Sex and Gender in Research

**Cell line source(s)**

Sf9 (Expression Systems; catalog #: 94-001F) and Tni (Expression Systems; catalog #: 94-002F) insect cells were used for baculovirus amplification and recombinant protein expression respectively.

**Authentication**

Cells have been authenticated by the vendors. No further authentication was performed for commercially available cell lines.

**Mycoplasma contamination**

Cells were not tested for mycoplasma contamination.

**Commonly misidentified lines (See ICLAC register)**

No commonly misidentified cell lines were used.
